# Tomato *SD1*, encoding a kinase-interacting protein, is a major locus controlling stem development

**DOI:** 10.1093/jxb/eraa144

**Published:** 2020-03-19

**Authors:** Jie Ye, Ranwen Tian, Xiangfei Meng, Peiwen Tao, Changxing Li, Genzhong Liu, Weifang Chen, Ying Wang, Hanxia Li, Zhibiao Ye, Yuyang Zhang

**Affiliations:** 1 The Key Laboratory of Horticultural Plant Biology, Ministry of Education, Huazhong Agricultural University, Wuhan, China; 2 Boyce Thompson Institute for Plant Research, Cornell University, Ithaca, NY, USA; 3 Fondazione Edmund Mach, Italy

**Keywords:** Domestication and improvement, GWAS, indel, kinase-interacting family protein, stem diameter, tomato

## Abstract

Stems serve as key determinants of plant development by connecting and supporting parts of the plant body, transporting nutrients important for long-distance communication that affect crop yield, and producing new organs. Nonetheless, studies on the regulation of stem development in crops are rather limited. Here, we found a significant correlation (*P*<0.001) between stem diameter (SD) and fruit size in tomato (*Solanum lycopersicum*). We performed a genome-wide association study and identified a novel quantitative trait locus (QTL), *SDR9* (*stem diameter regulator on CHROMOSOME 9*), that co-localized with a gene encoding a kinase-interacting family protein (KIP), which is the most likely candidate gene related to SD (hereafter referred to as *SD1*). Overexpression of *SD1* in thin-stem accessions resulted in increased SD. In contrast, suppressed expression of *SD1* in thick-stem accessions using RNA interference exhibited the opposite effect. Further microscopic analyses showed that *SD1* affected the stem diameter by controlling the size and number of secondary phloem cells. An 11-bp indel in the promoter region of *SD1* that disrupts a gibberellin-responsive *cis*-element was linked to SD. Expression analysis revealed that *SD1* was mainly expressed at the cambium of the stem and positively regulates stem development. Evolutionary analysis revealed that the thick-stem allele of *SD1* was selected during the recent process of tomato improvement. Our results provide novel genetic and molecular insight into natural variation of SD in tomato and may accelerate the breeding of high yield tomato.

## Teaser

*SD1*, mainly expressed at the cambium and selected during tomato improvement, encodes a kinase-interacting protein that positively regulates stem development by controlling the size and number of secondary phloem cells.

## Introduction

The flexibility of plant growth is striking, especially for stems, which are extremely variable in their size, architecture, and function (Sanchez *et al.*, 2012). Regulation of stem development is essential for this variability because stems perform essential roles for higher plants such as supporting and connecting various body parts, which allows for the transport of substances (e.g. water, nutrients, and signaling molecules) that are important for long-distance communication and affect crop yields (Sieburth and Deyholos, 2006; Dettmer *et al.*, 2009). The mature stem typically has two types of tissues that contribute to conduction—xylem (which transports water and dissolved minerals) and phloem (which distributes photosynthate and other molecules, such as RNA and proteins, from source to sink organs) (Dettmer *et al.*, 2009; Miyashima *et al.*, 2013). Mature stems also have undifferentiated procambial cells that serve as a reticulate system of meristematic cells and have the potential to produce vascular tissues (Ye, 2002).

To date, our knowledge of cell organization and differentiation of stem development has been well characterized in Arabidopsis and woody species (Sanchez *et al.*, 2012; Pierre-Jerome *et al.*, 2018). However, the regulation of stem augmentation in horticultural crops, such as tomato, involves some novel mechanisms (MacAlister *et al.*, 2012). For instance, the Arabidopsis *SHOOT MERISTEMLESS* (*STM*) is a well-characterized regulator of shoot apical meristem (SAM) and compound leaf maintenance (Aguilar-Martínez *et al.*, 2015; Roth *et al.*, 2018). In Arabidopsis, the *WUSCHEL* (*WUS*) gene positively regulates the maintenance of stem cell populations in shoot and floral meristems (Ikeda *et al.*, 2009), while the *CLV* genes (*CLV1*, *CLV2*, and *CLV3*) function to restrict the proliferation of these cells (Clark *et al.*, 1997; Somssich *et al.*, 2016). Moreover, the double mutant *wus clv* and single mutant *wus* show a similar phenotype in which the SAM arrests after the emergence of the first leaf, suggesting *wus* is almost completely epistatic to *clv* (Brand *et al.*, 2000). The mutual regulation between the positive *WUS* pathway and the negative *CLV* pathway provides a feedback system for maintaining the delicate balance required for proliferation of stem cells to proceed at the right time and in the right place (Brand *et al.*, 2000; Yadav *et al.*, 2013).

Cell size and the number of cell layers in the vascular tissues of a stem are key determinants of the stem thickness (Sanchez *et al.*, 2012). The Arabidopsis cyclin proteins AtcycD2;1 and AtE2Fa-DPa regulate stem development by promoting the transition from G1 to S phase in the cell cycle, thereby influencing cell division and proliferation in tobacco (Qi and John, 2007; Fujii *et al.*, 2012). Overexpression of two CCCH zinc finger genes, *AtTZF2* and *AtTZF3*, altered the pattern of plant growth, increased stem diameter, delayed senescence and enhanced longevity (Lee *et al.*, 2012). In important food crops, such as rice and wheat, forward genetic methods have also been employed to identify important QTLs involved in stem thickness traits linked to lodging resistance and yield (Kashiwagi and Ishimaru, 2004). Using backcross populations and near-isogenic lines (NILs), Ookawa *et al.* (2010) cloned *ABERRANT PANICLE ORGANIZATION1* (*APO1*) in rice, which has a major effect on stem diameter. Moreover, the overexpression of *APO1* led to a 20% increase in flower numbers per panicle and therefore increased yield. Similar research on the relationship between stem and yield was reported in wheat, soybean, and other crops (Hai *et al.*, 2005).

Phytohormones, including cytokinin, auxin, ethylene, brassinosteroids, abscisic acid, and gibberellin (GA), play important roles in SAM and stem development (Etchells *et al.*, 2012; Lee *et al.*, 2012; Vahala *et al.*, 2013; Azizi *et al.*, 2015; Best *et al.*, 2016; Krogan *et al.*, 2016; Shu *et al.*, 2016; Landrein *et al.*, 2018; Wang and Jiao, 2018). For GA, previous studies have shown that its biosynthesis occurred in the expanding xylem and regulated the early xylem differentiation and cell elongation in woody plants (Israelsson *et al.*, 2005). Exogenous GA induces the expression of both *NST1* and *NST3* (two key regulators related to the formation of secondary walls in the xylem of Arabidopsis) and regulates the secondary wall thickening of xylem fibers in woody plants (Mitsuda *et al.*, 2007; Ikematsu *et al.*, 2017). VASCULAR-RELATED NAC-DOMAIN (VND) 6 and 7 are plant-specific NAC-domain transcription factors that serve as transcriptional switches for plant metaxylem and protoxylem vessel formation, respectively (Kubo *et al.*, 2005).

There have been many receptor-like kinases (RLK) identified in a variety of plant species that been shown to play diverse physiological roles (Becraft, 1998, 2002; Torii and Clark, 2000; Vaid *et al.*, 2013). However, the identification and functional analysis of kinase-interacting proteins (KIPs) are largely lacking. The first plant *KIP* gene was demonstrated to bind to and be phosphorylated by PRK1 (a pollen-expressed receptor-like kinase of petunia) in *Petunia inflata* (Skirpan *et al.*, 2001). Using a high-throughput multiplexed assay, Jayaraman *et al.* (2017) identified a KIP1-like protein (Medtr5g032060) that is potentially targeted by lysin motif domain-containing receptor-like kinase 3 (LYK3) and is involved in the signal transduction that promotes the development of symbiotic systems in *Medicago*.

In this study, we performed association mapping with diverse tomato accessions collected from around the world using high-density genotype (Ye *et al.*, 2017) and SD phenotype data generated under multiple environments. Based on these data, we identified the first major gene underlying stem development, *SD1*, and we functionally and evolutionarily characterized it. Our data provide evidence that *SD1* was an improvement rather than domestication target. Furthermore, we found that the yield potential was improved in thick-stem accessions of cultivated tomato harboring *SD1*^TK^ (thick-stem genotype *SD1*).

## Materials and methods

### Plant materials and phenotyping

**Fig. 1.**
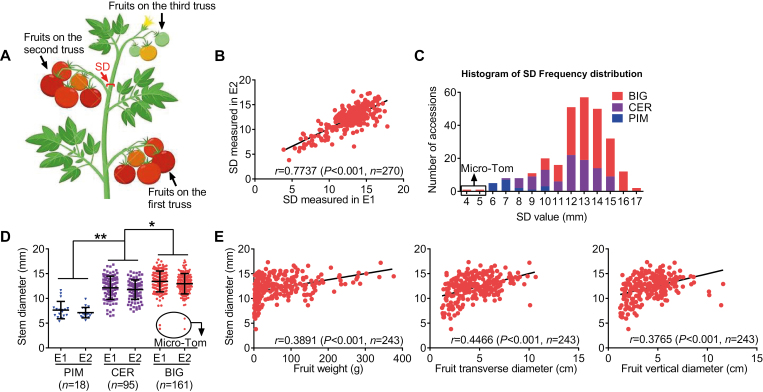
Generation and variation of phenotypic data. (A) Diagram showing the stem diameter (SD) measured in the study. (B) Correlation between SD values in plants from the collection of 270 tomato accessions grown in two different environments (E1 and E2). (C) Frequency distribution of SD in the three subgroups of the GWAS population. The arrow indicates two Micro-Tom accessions that belong to the BIG subgroup with small SD values. (D) Phenotypic variation of SD among the three subgroups of the GWAS population. The arrow indicates two Micro-Tom accessions. (E) Correlation between SD and fruit weight (left), fruit transverse diameter (middle), and fruit vertical diameter (right) in the natural population.

The 270 tomato accessions used in this study belonged to *Solanum pimpinellifolium* (PIM), *S. lycopersicum* var. *cerasiforme* (CER), and *S. l. lycopersicum* (BIG) (see Supplementary Table S1 at *JXB* online). This panel of tomato accessions was selected from a panel of 360 accessions that was previously described (Lin *et al.*, 2014). The experimental population was grown in Wuhan, China, in two locations in 2013: open-field cultivation at Huazhong Agricultural University (location of environment 1 (E1)) and greenhouse cultivation at Zhongdou Seed Company (location of environment 2 (E2)) as described previously (Ye *et al.*, 2017). For each accession, 12 plants were grown in a randomized complete-block design (including two rows of each accession and six plants in each row). For each accession, SD was measured on six plants randomly chosen among those having normally developed stems and fruits. For the genome-wide association study (GWAS) of stem diameter, the SD was measured using Vernier calipers at the second fruit truss when the fruit began ripening on the third fruit truss (Fig. 1A). For fruit size analysis, the fruits that appeared developmentally equivalent were harvested at red ripe stage (about 44 d after fertilization for most accessions). Three fruit size-related traits including fruit weight (investigated with an analytical balance), fruit transverse diameter, and vertical diameter (investigated with Vernier calipers) were measured for at least 10 ripe fruits per line, on a total of 243 accession (Supplementary Table S2).

### Genome-wide association study

A total of 4 180 023 single nucleotide polymorphisms (SNPs; minor allele frequency ≥0.05; the number of accessions with minor alleles ≥6) were used in the GWAS for SD in two different environments in the population as described previously (Ye *et al.*, 2019*a*). Detailed information on the SNPs used is available at the Sol Genomic Network (https://solgenomics.net/). The GWAS was performed using a compressed mixed linear model (MLM) (Yu *et al.*, 2006) with TASSEL 4.0 (Bradbury *et al.*, 2007), as described in a previous study (Ye *et al.*, 2017). The *P*-value of each SNP was calculated and defined as suggestive (1/*n*, ≤2.4×10^−7^) or significant (0.05/*n*, ≤1.2×10^−8^; where *n* is the total number of markers used). We used Haploview software to prepare a linkage disequilibrium (LD) heatmap surrounding the candidate gene on Chr09 in the GWAS (Barrett *et al.*, 2005). The physical locations of the SNPs were identified based on the tomato genomic sequence version SL2.50 (http://solgenomics.net/).

### DNA sequencing

To detect variation in the *SD1* gene region (chromosome 9: 68 234 000–68 243 700 bp; release SL2.50), DNA sequences of *SD1* in 10 thick-stem accessions (TS-22, TS-52, TS-72, TS-95 TS-111, TS-125, TS-143, TS-155, TS-179, and TS-193) and 10 thin-stem accessions (TS-16, TS-18, TS-37, TS-54, TS-79, TS-96, TS-227, TS-258, TS-267, and TS-291) were amplified using PCR with specific primers (see Supplementary Table S3). Because *SD1* is longer than 9700 bp, we used a piecemeal amplification strategy and amplified the gDNA fragment containing *SD1* in four overlapping segments. The PCR products were sequenced and compared with the reference genome to detect polymorphisms. In addition, DNA sequence polymorphisms in *SD1* for 240 tomato accessions (133 BIG accessions, 88 CER accessions and 19 PIM accessions; Supplementary Table S4) were downloaded from the public database (NCBI BioProject site under the accession PRJNA353161). Genotype analysis of Indel_11 of *SD1* was performed using molecular markers designed in the present study (see Results, Supplementary Fig. S4).

### Gene cloning, vector construction, and transformation

For transgenic function analysis, three accessions were selected as wild-type (WT): TS-9 (Ailsa Craig; AC), a BIG accession with thick stem, which was transformed with the RNA interference (RNAi) construct, and TS-18 (LA1579)/TS-19 (LA1589), two PIM accessions with thin stem, which were transformed with the overexpression constructs. For the *SD1*-RNAi construct, a 1028-bp fragment from the promoter of *SD1* was amplified using specific primers (see Supplementary Table S3) and then cloned into pHGRV using BP Clonase according to the manufacturer’s instructions (Thermo Fisher Scientific, USA). For the overexpression constructs, the complete *SD1* open reading frame was amplified from tomato cDNA (AC for *SD1*^TK^ and LA1589 for *SD1*^TN^) and then cloned into the pHELLSGATE8 vector using homologous recombination (ClonExpress II One Step Cloning Kit; Vazyme, Miramar Beach, FL, USA). For the Pro_*SD1*_::GUS construct, an upstream genomic DNA sequence (from −3088 to −1 bp) of *SD1* was amplified using sequence-specific primers. The fragment was recombined into pMV2 (modified from pHELLSGATE8) containing the β-glucuronidase (GUS) coding sequence. *Agrobacterium* strain C58 was transformed with all of these recombinant constructs using electroporation. Subsequently, these *Agrobacterium* strains were used to transform tomato cotyledon explants as described previously (Jones *et al.*, 2002). Transgenic plants were identified using PCR with a CaMV35S promoter forward primer and a *SD1-*specific reverse primer.

### Sample preparation, RNA isolation, and gene expression analysis

For RNA isolation, the young stem close to the SAM was sampled when the fruits on the third truss began to ripen. Different tissues of WT (AC and LA1589) and transgenic plants were also sampled for expression profiling analysis of *SD1*. Total RNA was extracted from the SD diversity accessions (AC and LA1589) and the transgenic lines using the TRIzol reagent (Thermo Fisher Scientific, USA). The cDNAs were synthesized from the total RNA using HiScript®II Reverse Transcriptase (Vazyme) following the manufacturer’s protocol. Gene expression was quantified using real-time quantitative RT-PCR (qRT-PCR) as previously described (Ye *et al.*, 2019*b*). The primer pair sequences (designed using Primer Premier 5.0) are listed in Supplementary Table S3. The *Actin* gene (Solyc11g008430) was used as an internal standard. The relative expression of specific genes was quantified using the comparative *C*_T_ method.

### Plant growth and GA treatment

Seeds (AC and LA1589) were sown individually in compost in plastic trays and grown in a photoperiod of 16 h light–8 h dark at 25 °C in a greenhouse. For the GA treatment, 1-month-old seedlings were sprayed with a 100 µM GA_3_ solution (GA_3_ dissolved in distilled water containing 0.1% Tween-20) or with distilled water containing 0.1% Tween-20 (control). Three young stems, from three independent plants of each genotype, were collected at 0, 1, 2, 4, 7.5, 12, and 24 h from the GA_3_-treated or control plants and stored under −80 °C for RNA isolation and expression quantification.

### Paraffin sections and fluorescence *in situ* hybridization

Light microscopic observation of paraffin sections was used to measure the number of cell layers and cell size in the stems of both WT and transgenic plants. For the stem paraffin sections, the stem was sampled at the second fruit truss (Fig. 1A) when the fruit began ripening on the third fruit truss. Paraffin sections were prepared as described by Yang *et al.* (2011). The number of cell layers and cell size were calculated manually. Photomicrographs were taken using an Olympus microscope. For *in situ* hybridization, a unique fragment of the *SD1* open reading frame (ORF) from 5338 to 5370 bp was selected as a template to synthesize a red fluorescent probe (5′-AACCUCUAGCUGCAACCGUCCUAUCUUCUCAG-3′). The tomato stem paraffin sections were processed as described by Sunde *et al.* (2003).

### GUS staining

Tomato samples from the transgenic lines harboring a transgene with the native promoter of *SD1* driving expression of the *GUS* gene (Pro_*SD1*_::GUS) were stained with a GUS staining solution (1 mM 5-bromo-4-chloro-3-indolyl-β-D-glucuronic acid (X-gluc), 10% methanol, 0.5% Triton X-100 and 50 mM NaPO_4_) to evaluate GUS activity. Staining was allowed to proceed for 12 h at 37 °C in the dark. Subsequently, the chlorophyll was removed using a graded ethanol series at room temperature. The specimens were observed using light microscopy with an Olympus SZX12.

### Molecular marker development for stem diameter in tomato

Six randomly selected tomato accessions including three thick-stem accessions (TS-125, TS-193, and TS-327) and three thin-stem accessions (TS-7, TS-123, and TS-226) were genotyped using PCR-based markers. PCR was performed to amplify either a 500-bp or a 700-bp fragment from the functional *SD1*^Indel_11^ sequence using specific primers (see Supplementary Table S3). The PCR program was conducted with four different annealing temperature as follows: (i) 3 min at 94 °C; (ii) 34 cycles of 30 s at 94 °C, 30 s at 56 °C/55 °C/54 °C/53 °C, and 20 s at 72 °C; and (iii) 10 min at 72 °C. The PCR products were then separated by electrophoresis in 1% agarose gels stained with ethidium bromide and visualized using UV light.

### Molecular diversity analysis

For the molecular diversity analysis, the π ratios and *F*_st_ were used to identify the selective sweeps in *SDR9* associated with tomato domestication and improvement events. Briefly, π (π _PIM_, π _CER_, and π _BIG_) and *F*_ST_ (*F*_ST_ (PIM/CER) and *F*_ST_ (CER/BIG)) were calculated using DnaSP5.0 version 5.0 (Librado and Rozas, 2009) and Genepop 4.2.2 (Rousset, 2008), respectively, with a sliding window length of 100 bp and a step size of 25 bp.

### Statistical analyses

The values for the coefficients of variation for stem diameter were estimated as σ/μ, where σ and μ are the standard deviation and mean values of the stem diameter in the population, respectively. Broad-sense heritability (*H*^*2*^) was estimated using the following equation: *H*^*2*^=Var_G_/(Var_G_+Var_E_), where Var_G_ and Var_E_ are genetic variance and environmental variance, respectively. Differences in stem diameter among the three subgroups (PIM, CER, and BIG) were analysed using an ANOVA. *P*<0.05 and *P*<0.01 were considered to be statistically significant. Finally, the significance of correlations between stem diameter and fruit weight/fruit transverse diameter/fruit vertical diameter were analysed using an *F*-test. *P*<0.01 was considered to be statistically significant.

## Results

### GWAS on stem diameter in tomato

Based on a phenotypic survey (described in ‘Materials and methods’), we observed consistent SD values between the two experimental environments (see Supplementary Table S1). The broad-sense heritability (*H*^*2*^) and coefficient of variation were 75.2% and 19.05%, respectively (Fig. 1B). This indicated that genetic factors rather than environmental factors were most important for determining SD. Among the subgroups, PIM tomato accessions had the narrowest SD, which varied from 5.77 to 10.45 mm with a mean of 7.55 mm (Fig. 1C, D). With the exception of two Micro-Tom accessions (TS-7 with an SD of 4.7 mm and TS-226 with an SD of 4.2 mm), the SD of the BIG tomato accessions (SD ranging from 8.65 to 16.65 mm with a mean of 13.33 mm) was significantly wider than that of both PIM and CER tomato accessions (SD ranging from 7.3 to 15.46 mm with a mean of 11.96 mm). A wide variation for SD in each subgroup provides evidence that SD was selected during tomato domestication and improvement. Furthermore, a significant correlation was observed between SD and the three fruit size traits, namely fruit weight, fruit transverse diameter, and vertical diameter (Fig. 1E). Thus, stem thickness is closely related to fruit size and potentially affects yield in tomato.

**Fig. 2.**
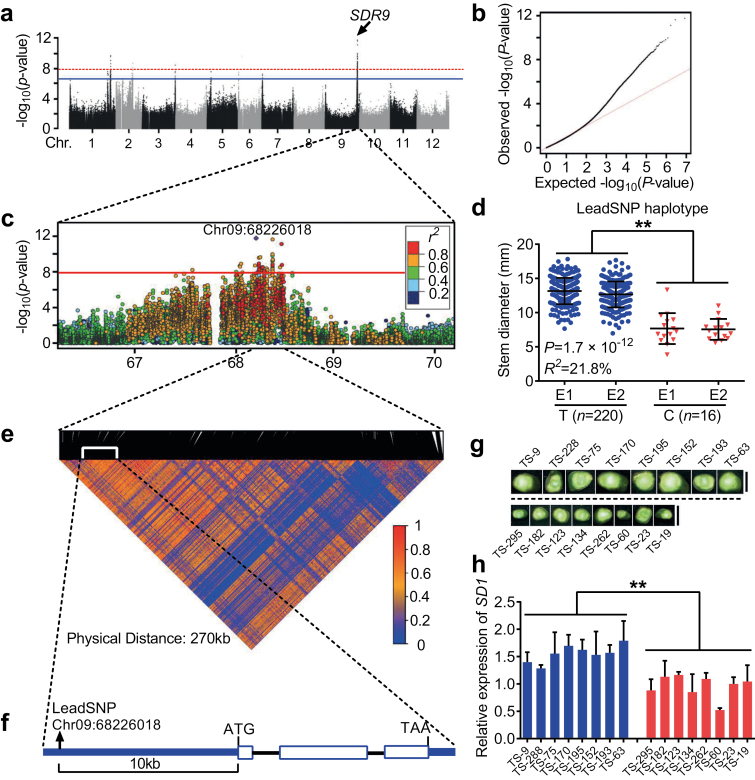
Genome-wide association study of the stem diameter (SD) trait in environment 1. (A) Manhattan plot displaying the genome-wide association signals for SD in the tomato genome (*n*=270) using the compressed MLM model. The *y-*axis indicates –log_10_ transformed observed *P*-values. The blue and red horizontal dashed lines indicate a genome-wide suggestive (2.4×10^–7^) and significant (1.2×10^–8^) threshold, respectively. The arrow indicates the most significant signal (*SDR9*) on chromosome 9. (B) Quantile-quantile plot for SD in the GWAS population. (C) Genome-wide association signal for the SD is shown on the 66.2–70.2 Mb region (*x*-axis) of chromosome 9. The lead SNP is indicated in purple and the colour of each plot corresponds to the *r*^*2*^ value (a measure of LD) according to the legend. (D) Box plot for SD in two genotypes distinguished by the lead SNP Chr09:68226018. (E) Representation of the pairwise *r*^2^ values (a measure of LD) among all polymorphic sites in the 270-kb genomic region corresponding to (C). The 145 haploblocks are represented by the inverted triangle. The haploblock contains the lead SNP associated with SD as indicated. (F) Gene structure of *SD1* (Solyc09g082510). The blue box, blue lines and black lines represent coding sequence, promoter and 3′-UTR, and introns, respectively. The relative position of the lead SNP to *SD1* is indicated. (G, H) SD (G) and the relative expression of the candidate gene (*SD1*) (H) in the stem apices of 16 selected accessions (eight thin-stem accessions and eight thick-stem accessions). Data represent means ±standard deviation (*n*=3). Asterisks indicate significant differences by *t*-test: **P<0.01.

We performed a GWAS using a compressed MLM with whole-genome diverse SNPs to reveal the genetic mechanism responsible for tomato SD in different environments (E1 and E2). The Manhattan and quantile–quantile plots produced with these GWAS data (Fig. 2; Supplementary Fig. S1) indicate that nine SNPs (*P*<2.4×10^−7^) are associated with SD in the two environments (see Supplementary Table S5). These SNPs are located on chromosomes 1, 2, 3, 5, 6, 7, 9, and 10, indicating that at the fruit ripening stage, stem development in tomato is controlled by multiple genes. Among the nine SNPs, two SNPs (SL2.50ch01_87168247 with *P*=2.0×10^−10^ and SL2.50ch09_68226018 with *P*=1.7×10^−12^) were significantly associated with tomato SD and could be repeatedly detected in different environments (E1 and E2). The lead SNP with the highest association to SD was SL2.50ch09_68226018 (*P*=1.7×10^−12^). This SNP explained 21.8% of the total variance observed in tomato SD. These data indicate that a candidate gene associated with the lead SNP is the major genetic locus responsible for the natural variation in tomato SD. We therefore named this locus *stem diameter regulator on CHROMOSOME 9* (*SDR9*). Two major haplotypes based on the lead SNP (SL2.50ch09_68226018, T/C) from the association signal—thick-stem haplotype (TkSH, T) and thin-stem haplotype (TnSH, C)—were associated with thick-stem and thin-stem phenotypes in tomatoes, respectively (Fig. 2D).

Within *SDR9*, we observed 47 SNPs that were significantly associated with SD (*P*<1.2×10^−8^) (see Supplementary Table S6), and there were a total of 21 genes within the 100-kb sequence on either side of SNP SL2.50ch09_68226018 (Supplementary Table S7). We then analysed the pairwise LD distance within the 4-Mb interval centered on the lead SNP (SL2.50ch09_68226018) from the GWAS. With the exception of six SNPs (SL2.50ch09_67583603, SL2.50ch09_68026123, SL2.50ch09_68061988, SL2.50ch09_68064331, SL2.50ch09_68074797, and SL2.50ch09_68087793), all significant SNPs (*P*<1.2×10^−8^) fall in a 270 kb region from 68.21 to 68.48 Mb (Fig. 2C). A haploblock (SL2.50ch09_68225271–SL2.50ch09_68245863) containing the lead SNP (SL2.50ch09_68226018) was identified from a haplotype analysis of the region spanning all of the significant SNPs on chromosome 9 (270 kb) (Fig. 2E). The haploblock (20.5592 kb) contains only one gene, a kinase-interacting family protein gene (*KIP*: Solyc09g082510, 68 236 188–68 243 303 bp). The lead SNP (SL2.50ch09_68226018) is located 10 kb upstream of the *KIP* CDS in the promoter region (Fig. 2F). These data provide evidence that *KIP* could contribute to stem development. To test whether KIP has a key regulatory role in the development of SD, we randomly selected eight accessions with thick-stem phenotypes and eight accessions with thin-stem phenotypes and quantified the expression of *KIP* in the stem using quantitative RT-PCR (Fig. 2G). The expression of Solyc09g082510 was significantly higher in the thick-stem accessions relative to the thin-stem accessions (Fig. 2H). Based on these results, we concluded that the Solyc09g082510 gene (hereafter referred to as *SD1*) is probably *SDR9*.

### *SD1* haplotypes in the GWAS population

*SD1* contains three exons and two introns and encodes a protein containing 1860 amino acid residues with three conserved domains including the KIP1 domain, Smc domain and SMC_prok_B domain (see Supplementary Fig. S2). Consistent with a large number of *KIP* genes in Arabidopsis (12 *KIP* genes) and rice (10 *KIP* genes) there are 11 *KIP* genes in the tomato genome. The amino acid sequence of SD1 is most similar to Networked 1A (NET1A) (40%) and Networked 1B (NET1B) (38%) from Arabidopsis (Supplementary Fig. S2C, Supplementary Dataset S1).

**Fig. 3.**
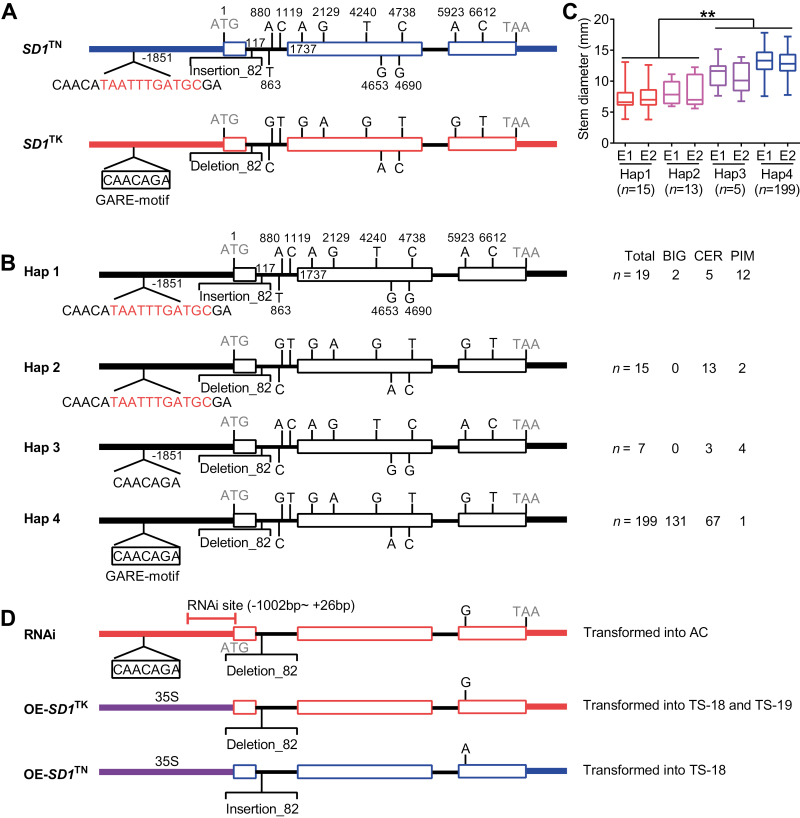
Haplotype analysis of *SD1* in tomato. (A) Structural variations of *SD1* in thin-stem and thick-stem accessions. The heavy lines represent the promoter and 3′-UTRs. The boxes represent coding sequences. The fine lines between the white boxes represent the introns. The 13 nucleotide polymorphisms including 11 SNPS and two indels are indicated by their positions. (B) Structural variations of the four *SD1* haplotypes in 240 tomato accessions. (C) Stem diameter of four *SD1* haplotypes. (D) Constructs used for transformation. The RNAi vector was constructed using the indicated fragment. The OE-*SD1*^TK^ and OE-*SD1*^TN^ vectors were construct using the CaMV35S promoter (*35S*_pro_) driving expression of the 5.6-kb ORF from AC (a thick-stem accession) and LA1589 (a thin-stem accession).

Based on a GWAS and both LD and expression analyses, *SD1* is likely the candidate responsible for the variation in SD in the natural population of tomato. To determine the DNA sequences responsible for the allelic variation in *SD1*, we sequenced the genomic region surrounding *SD1*, including the promoters, exons, introns, and 3′-untranslated regions (UTRs) in 10 thick-stem accessions and 10 thin-stem accessions. We found that *SD1* can be classified into two different haplotypes, *SD1*^TK^ for the thick-stem phenotypes and *SD1*^TN^ for the thin-stem phenotypes (Fig. 3A). Comparative analysis of the *SD1*^TK^ and *SD1*^TN^ sequences showed that a total of 13 polymorphisms were tightly associated with the lead SNP (SL2.50ch09_68226018) (see Supplementary Table S4), including two indels (Indel_11, TAATTTGATGC from position −1861 to −1851; Indel_82 in the first intron from position −117 to −198), three SNPs (SNP1, C>T variant at position 863; SNP2, G>A variant at position 880; and SNP3, T>C variant at position 1119) in intron 1, and eight exon SNPs including seven non-synonymous SNPs (SNP4, G>A variant at position 1737; SNP5, A>G variant at position 2129; SNP6, G>T variant at position 4240; SNP7, A>G variant at position 4653; SNP8, C>G variant at position 4690; SNP9, T>C variant at position 4738; SNP11, T>C variant at position 6612) and one non-synonymous polymorphism (SNP10, G>A variant at position 5923, causing an amino acid change from V to I). Furthermore, the larger natural population of 240 tomato accessions was classified into four different haplotypes (Hap 1, Hap 2, Hap 3, and Hap 4), based on these 13 polymorphisms (Fig. 3B). Interestingly, except two Micro-Tom accessions (TS-7 and TS-226) that belong to the BIG group, Hap 1 mainly consists of PIM and CER accessions with the lowest SD values. Hap 2 and Hap 3, composed of only PIM and CER, showed lower SD than Hap 4, which mainly consists of BIG accessions (Fig 3B, C).

Haplotype and expression analysis of *SD1* in the diverse SD accessions was consistent with Indel_11, the only polymorphism in the promoter region of *SD1*, which potentially regulate its expression (Figs 2H, 3). Interestingly, Indel_11 in the promoter region of *SD1* led to the formation of a GARE motif (CAACAGA), a (GA)-responsive element that activates the transcription of downstream target genes (Xie *et al.*, 2016). Given that extensive studies have established that GAs control stem development (Xu *et al.*, 1997; King *et al.*, 2001; Plackett *et al.*, 2011), we thought that Indel_11 was probably the genetic basis of the variance of *SD1* expression and stem development.

### *SD1* positively regulates stem diameter and fruit size

**Fig. 4.**
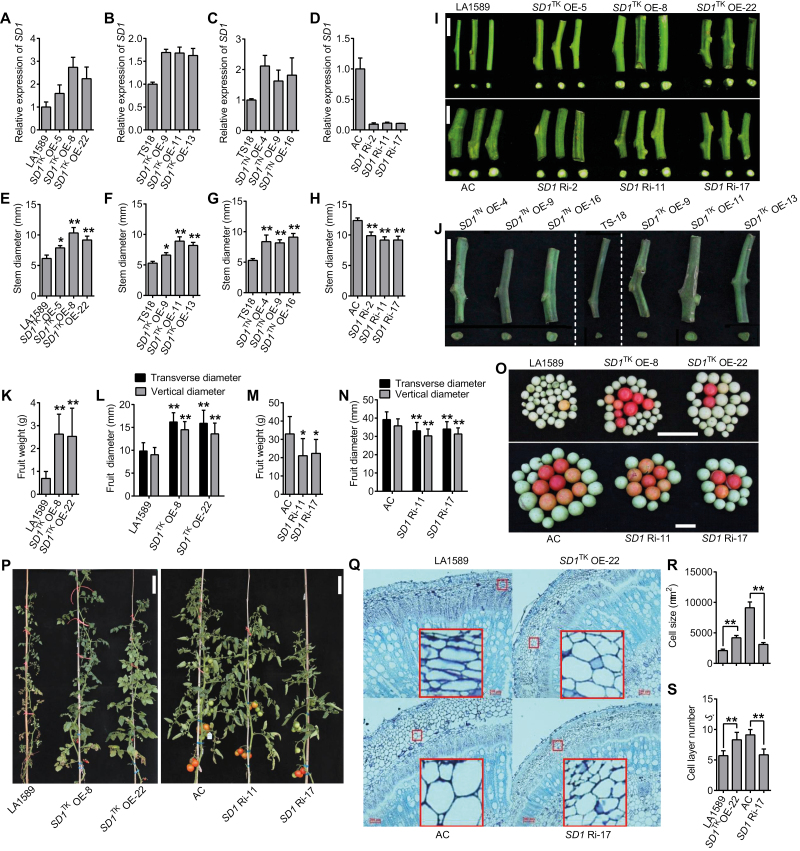
Manipulating the expression of *SD1* altered stem diameter and fruit size of tomato. (A–D) Relative expression of *SD1* in *SD1*^TK^/LA1589 overexpressing (A), *SD1*^TK^/TS-18 overexpressing (B), *SD1*^TN^/TS-18 overexpressing (C), and *SD1* RNAi (D) transgenic tomato lines. (E–H) Stem diameter in *SD1*^TK^/LA1589 overexpressing (E), *SD1*^TK^/TS-18 overexpressing (F), *SD1*^TN^/TS-18 overexpressing (G), and *SD1* RNAi (H) transgenic tomato lines. (I) Comparison of the stem thickness for wild-type (WT; LA1589 and AC) and the transgenic lines with the *SD1*^TK^ overexpressing and *SD1* RNAi transgenes, respectively. Scale bars, 2 cm. (J) Comparison of stem thickness for WT TS-18 and the transgenic lines that overexpress *SD1*^TK^ and *SD1*^TN^. Scale bars, 2 cm. (K–N) Fruit weight (K, M) and transverse/vertical diameter (L, N) in *SD1*^TK^ overexpressing (K, L) and *SD1* RNAi (M, N) lines relative to WT (LA1589 and AC). (O, P) Comparison of the fruit size (O) and the plant morphologies (P) of WT (LA1589 and AC) and *SD1*^TK^ overexpressing and *SD1* RNAi transgenic lines, respectively. Scale bars, 5 cm (O) and 20 cm (P). (Q) Comparison of cross-sections of young stems from *SD1*^TK^ overexpressing and *SD1* RNAi lines with WT (LA1589 and AC). Scale bars, 200 µm. (R, S) cell size (R) and number of layers (S) of parenchyma cells in the young stems of *SD1*^TK^ overexpressing and *SD1* RNAi lines relative to WT (LA1589 and AC). Data in the graphs are presented as means ±SEM. Asterisks indicate statistically significant differences calculated with Student’s *t*-test: *P<0.05, **P<0.01.

To further functionally characterize the role of Indel_11 and SNP10 in the development of SD, we generated transgenic plants using three different constructs. We transformed TS-18 (LA1579) and TS-19 (LA1589) (two thin-stem accessions in PIM) with a construct that overexpresses the *SD1*^TK^ allele (Fig. 3D). We also transformed TS-18 with a construct that overexpresses the *SD1*^TN^ allele (Fig. 3D). We transformed AC (a thick-stem accession from the BIG group) with an *SD1* RNAi construct (Fig. 3D). Both *SD1*^TK^ and *SD1*^TN^ overexpressing (OE) transgenic plants developed comparable and significant increases in SD compared with WT. Consistently, knocking-down of the expression of *SD1* with the RNAi construct significantly reduced the SD compared with AC (Fig. 4A–J). These results indicate that *SD1* is a positive regulator to tomato stem development and that both the SNP10^A^ allele (Hap 1 and Hap 3) and the SNP10^G^ allele (Hap 2 and Hap 4) are functional. Furthermore, we observed significant increases and decreases in fruit size in the *SD1* overexpressing and RNAi plants, respectively. These data provide evidence that *SD1* potentially affects fruit size by regulating stem development (Fig. 4K–P). All of these results make the case that the nucleotide differences in the promoter region of *SD1* and presence or absence of the GARE motif are mostly responsible for the natural variation in SD and QTL *SDR9*.

Moreover, we developed a co-dominant molecular marker based on the *SD1*^Indel_11^ polymorphism for indirect selection of stem thickness in tomato plants. Indel_11 falls on the 3′ end of the forward primer, and the reverse primer was located 500 bp downstream of Indel_11 (see Supplementary Fig. S3). There was a repeated sequence containing the forward primers located 200 bp upstream of Indel_11. Thus, amplification of DNA from the thick-stem accessions with a deletion of 11 bp yields a 700 bp band. In contrast, amplification of DNA from the thin-stem accessions with an insertion of 11 bp yields a 500-bp band. Thus, this marker can distinguish tomato accessions with large variations in SD.

To better understand the role of *SD1* in the regulation of SD, we studied the cell number and cell size in stems using paraffin sections. The *SD1* overexpressing lines showed significantly increased cell size and cell layers in stem parenchymal cells compared with LA1589 (Fig. 4Q–S). Consistently, we observed that the size and the number of layers of parenchymal cells significantly decreased in the RNAi lines compared with AC. Interestingly, the number of cell layers of secondary xylem and secondary phloem decreased in the stems of the RNAi lines compared with AC, but no significant changes were observed in the stems of the overexpression lines compared with LA1589 (see Supplementary Fig. S4). These results indicate that the increase in SD associated with *SD1* might result mainly from both cell expansion and increases in cell number in the parenchyma tissue of stem.

### Expression pattern of *SD1* in thick- and thin-stem genotypes

**Fig. 5.**
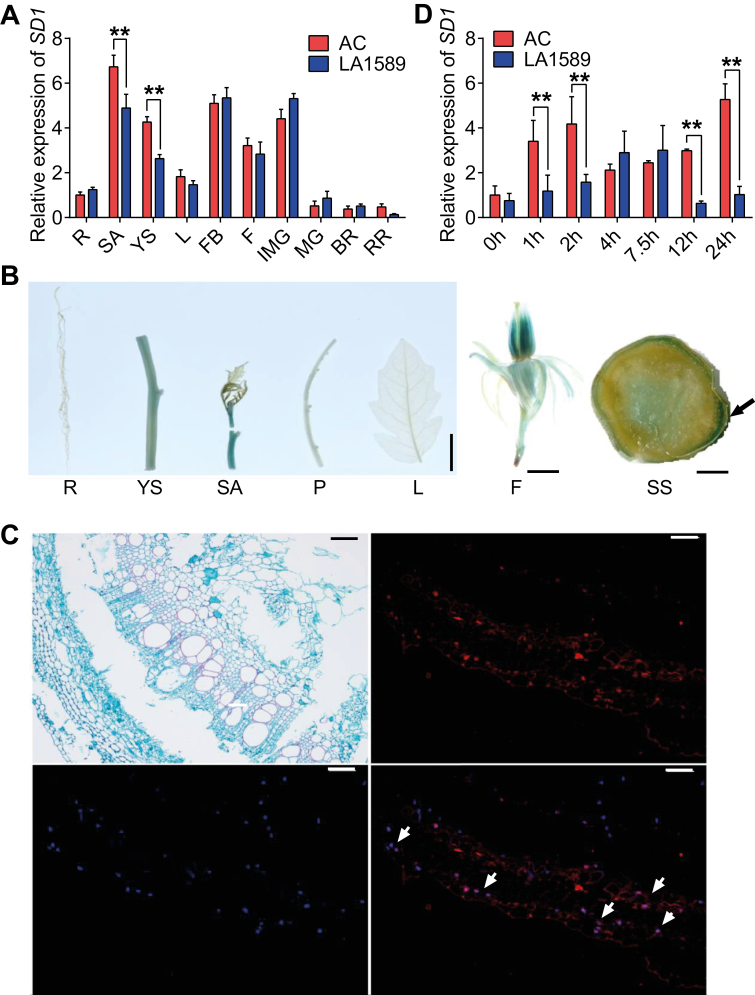
Tissue-specific and induced expression analysis of *SD1* in tomato. (A) Transcript levels of *SD1* in different tomato organs: BR, breaker stage fruit; F, flower; FB, flower bud; IMG, immature fruit; L, leaf; MG, mature green fruit; R, root; RR, red ripe stage fruit; SA, stem apex; YS, young stem. AC is a thick-stem accession. LA1589 is a thin-stem accession. (B) Histochemical localization of GUS activity in different tomato organs of transgenic plants harbouring Pro_*SD1*_::GUS. F, flower; L, leaf; P, petiole; R, root; SA, stem apex; SS, stem cross-section; YS, young stem. The arrow indicates high expression of *SD1* in the cross section of the stem. Scale bars, 3 cm (left), 2 cm (middle), and 0.5 cm (right). (C) *In situ* hybridization of *SD1* in a cross section of the young stem. Scale bars, 200 µm. The arrow indicates high expression of *SD1* in the stem cross section. (D) Expression of *SD1* under exogenous GA_3_ treatment in young stems from wild type (LA1589 and AC). Data in the graphs are presented as means ±SEM. Asterisks indicate statistically significant differences calculated with Student’s *t* test: **P<0.01.

To investigate the spatial and temporal expression patterns of *SD1*, qRT-PCR was performed to detect the expression levels of *SD1* in different tissues and during fruit development in thick-stem (AC) and thin-stem (LA1589) accessions (Fig. 5A). *SD1* was expressed at high levels in young tissues including stem apices, young stems, flower buds, flowers, and young fruit, but was expressed at low levels in roots, leaves, and mature fruit. *SD1* was expressed at significantly higher levels in the stem tissues of AC compared with LA1589, consistent with its role for *SD1* in positively regulating stem development in tomato. The spatiotemporal expression of *SD1* was further studied by monitoring GUS activity in the different tissues of transgenic lines harboring the Pro_*SD1*_::GUS reporter gene (Fig. 5B). Consistent with the results from our experiments that utilized qRT-PCR, we observed high GUS activity in the stem apex, young stem, and flower. GUS activity was mostly detected in the cambium of young stems. To better visualize the expression pattern, the expression of *SD1* in the young stem was investigated using *in situ* hybridization. The purple fluorescence observed by merging the blue fluorescence emitted by the 4′,6-diamidino-2-phenylindole stain and the red fluorescence emitted by the *SD1* probe was specifically detected at the cambium of the stem (Fig. 5C), which is consistent with GUS staining results for the young stem. Taken together, these results indicate that *SD1* is actively expressed in the cambium of the stem where *SD1* positively regulates stem development.

The above results showed that the presence or absence of the GARE motif in Indel_11 exerted a major influence on the expression of *SD1* and the variation of SD in tomato (Figs 2, 3). To assess whether GA is involved in *SD1*-mediated stem development, we analysed the expression response of *SD1* to exogenous GA_3_. Compared with LA1589 (*SD1* gene containing the GARE motif), GA-induced expression of *SD1* was more sensitive and stronger in AC (*SD1* gene lacking the GARE motif) (Fig. 5D). This expression pattern provides evidence that the varying degrees of *SD1* expression response to GA_3_ between thick- and thin-stem accessions could be attributed to absence or presence of the GARE motif in the *SD1* promoter.

### *SD1* ^Indel_11^ selection during tomato improvement

**Fig. 6.**
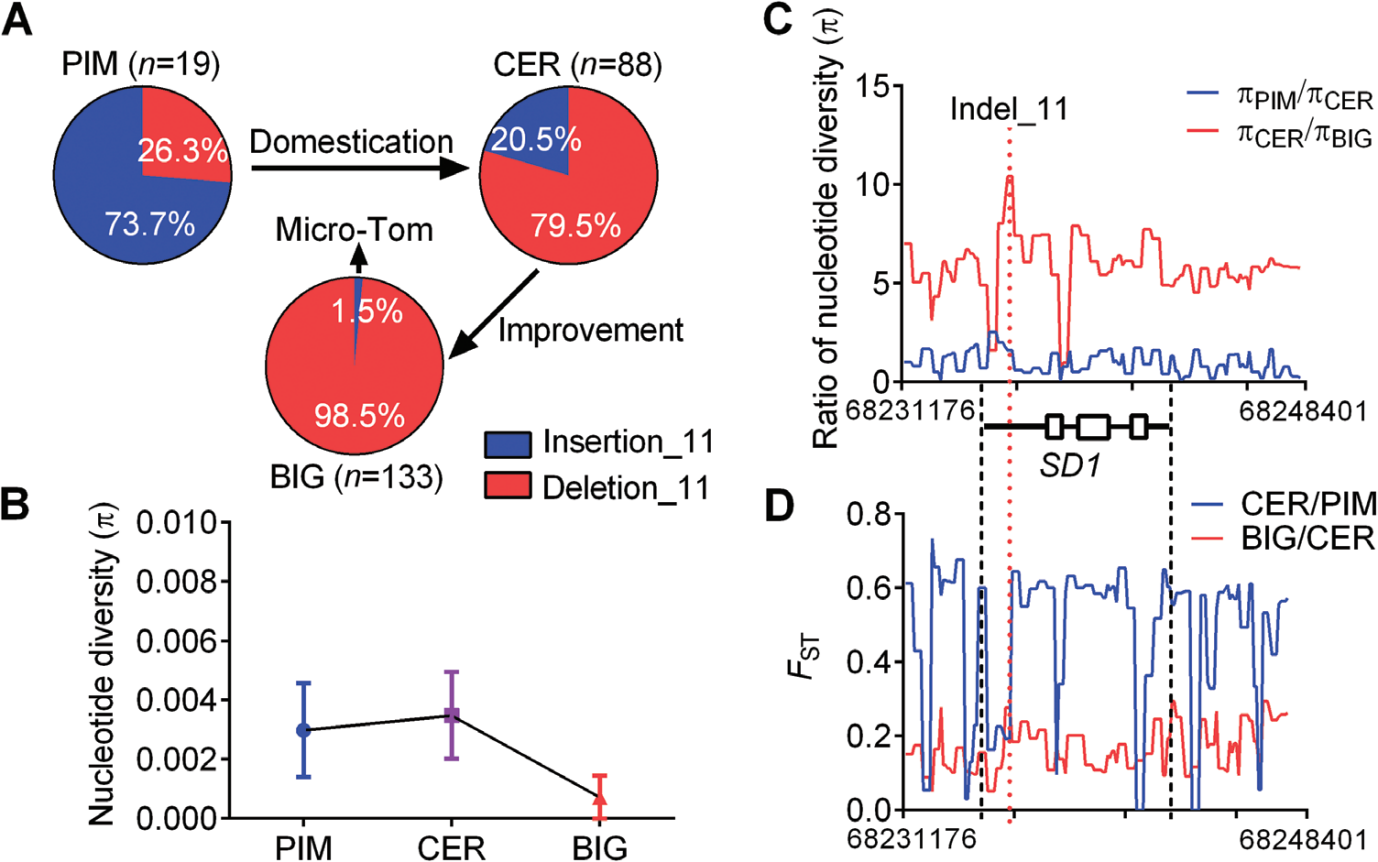
Nucleotide diversity of *SDR9* loci during tomato domestication and improvement. (A) Frequency of derived and ancestral Indel_11 allele in tomato subpopulations PIM, CER, and BIG. *n*, number of accessions. The arrow indicates that the only two accessions with Insertion_11 were Micro-Tom with thin stems. (B) Average nucleotide diversity (π) values for PIM (blue), CER (purple), and BIG (red line) within the 17-kb region (68 231 176–68 248 401) of *SDR9*. (C, D) The ratio of nucleotide diversity (π) (C) and genetic differentiation (*F*_ST_) (D) was calculated from the *SD1* sequences from the different subgroups. In total, 240 tomato accessions (refer to Supplementary Table S4) were used for the analysis, including 19 PIM accessions, 88 CER accessions, and 133 BIG accessions. The red dotted line indicates the location of Indel_11. The relative position of genomic DNA of *SD1* is indicated with black dotted lines.

Because Indel_11 influences the expression of *SD1* in the tomato stem, we genotyped the Indel_11 variants in a tomato collection that contains 133 BIG accessions, 88 CER accessions, and 19 PIM accessions and that covers the proposed trajectory of tomato domestication and improvement (Fig. 6A; Supplementary Table S8). A total of 74% (14 of the 19) of the PIM accessions carried Insertion_11. These data provide evidence that the Insertion_11 genotype of *SD1* was prevalent before tomato domestication and that the Deletion_11 genotype existed at an early period in the evolution of tomato. The allele frequency of Indel_11 increased moderately in the CER group and significantly increased in the BIG accessions (Fig. 6A). The only two accessions in the BIG group that carry Insertion_11 are two Micro-Tom accessions named TS-7 and TS-226. The two accessions have very thin stems with Insertion_11 genotype of *SD1*, which is consistent with our haplotype analysis (Fig. 3; Supplementary Table S8).

To examine the evolutionary history of the *SDR9* locus, DNA sequence variation in the genomic region including the coding region and 5-kb upstream and downstream of *SD1* was sequenced. In the *SD1* region, nucleotide diversity (π) in BIG (0.000718) was reduced relative to CER (0.003482) and PIM (0.002983) (Fig. 6B). Although the highest CER to BIG ratio of nucleotide diversity (π _CER_/π _BIG_, 10.42) surrounding *SDR9* appears at Indel_11 in the *SD1* promoter (Fig. 6C), the value of π _PIM_/π _CER_ was relatively low. These data are consistent with the *SDR9* loci contributing to improvement but not to a domestication event. This is consistent with a previous report arguing that *SD1* contributed to improvement but was not part of a domestication sweep at the whole genome level (Lin *et al.*, 2014). Furthermore, we computed the population differentiation statistic (*F*_ST_) for each SNP site surrounding *SDR9* for the genotyped population. We observed the most divergent site between the CER and BIG groups at the position of Indel_11 in the *SD1* promoter (Fig. 6D). Altogether, these data suggested that the derived allele of *SD1* occurred early before domestication but Deletion_11 in *SD1* only became relevant during the recent process of improvement.

## Discussion

Besides its importance in yield composition in woody plants (Génard *et al.*, 2001), SD is one of the key factors contributing to lodging resistance in cereal crops (Kashiwagi *et al.*, 2008). Few studies on molecular regulation of SD have been reported in tomato (Meng *et al.*, 2017; Zuo *et al.*, 2017). Here, using correlation analysis, we found that SD was significantly correlated with fruit size in a natural population of tomato (Fig. 1), providing evidence of the importance of SD in tomato yield. Subsequently, we identified a gene encoding a kinase-interacting protein that is probably the first domesticated gene related to stem diameter. We named this gene *SD1* and found that it is actively expressed in the cambium of the stem and positively regulates stem development in tomato (Fig. 5). Moreover, we found that fruit size and potential yield increased and decreased in *SD1* overexpression and RNAi transgenic plants, respectively (Fig. 4). This result supports the idea that *SD1* influence yield in tomato by directly regulating stem development.

The identification of genes for important agronomic traits that are subjected to domestication and improvement will help us to accelerate the process of domesticating new crops (Lemmon *et al.*, 2018; Li *et al.*, 2018). In this study, we found that the proportion of Deletion_11 in *SD1* dramatically increased during tomato domestication and improvement (26.3% of PIM accessions, 79.5% of CER accessions, and 98.5% of BIG accessions) (Fig. 6). These data suggested that Deletion_11 was selected for both tomato domestication and improvement. However, population differentiation (*F*_ST_) and nucleotide diversity (π) indicated the major selection of Deletion_11 occurred at the improvement step (Fig. 6C, D). Previous studies have shown that only 21% of the domestication sweeps overlapped with improvement sweeps in tomato, indicating that most improvement loci (79%) have undergone only one round of selection for the further improvement of agronomic traits after domestication (Lin *et al.*, 2014). The *SD1* regulating stem development in tomato conforms to one-step (improvement) selection. Interestingly, the *SDR9* loci physically overlapped with *fw9.3*, a fruit weight QTL on chromosome 9 (Lin *et al.*, 2014). The *fw9.3* QTL was localized within improvement sweeps that contributed to the second round of fruit enlargement during the CER-to-BIG transition, consistent with the improvement selection of *SDR9* loci in our study. Therefore, we hypothesize that *fw9.3* for fruit weight was selected during tomato improvement, and *SDR9* hitchhiked due its physical proximity in this process.

*SD1* encodes a kinase-interacting protein, and the proteins with the most similarity in Arabidopsis are NET1A and NET1B (see Supplementary Fig. S2). NET1A, which localizes to the microfilament and is expressed in root meristems and throughout the mature vasculature, potentially couples different membranes to the actin cytoskeleton in plant cells and mediates actin–membrane interactions (Deeks *et al.*, 2012). Primary root growth is enhanced in the double mutants of *NET1A* and *NET1B*, which leads to a significant acceleration of root-cell expansion (Deeks *et al.*, 2012). *SD1*, mainly expressed at the cambium of stem, enhanced stem thickening by accelerating stem cell expansion and division in tomato (Fig. 4Q–S). Given that kinase-interacting protein similarly regulates cell enlargement in Arabidopsis and tomato, it probably contributes to stem and root development in other crops. In Arabidopsis, the *PXY* (*P*hloem intercalated with *Xy*lem) gene encodes a receptor-like kinase that plays an essential role in maintaining the cell polarity that is required for cell division during stem vascular development (Fisher and Turner, 2007). To further refine our understanding of the *SD1*-dependent molecular mechanism that contributes to stem development of tomato, it would be interesting to identify tomato RIKs that are functionally similar to PXY.

In this work, we proved that variation in transcript abundance caused by Inddel_11, rather than variation in the protein-coding sequence (SNP10^A/G^), in *SD1* was responsible for the *SD1*-dependent SD variation in tomato (Figs 3, 4). This finding is consistent with our previous reports that attributed natural variation in tomato fruit malate and ascorbate content to expression variation in *SlALMT9* and *SlbHLH59*, respectively (Ye et al., 2017, 2019*a*). Transgenic complementation tests with two *SD1* constructs, *SD1*^TK^ and *SD1*^TN^, showed that thin-stem accessions (TS-18 and TS-19) can be complemented by overexpressing either the *SD1*^TK^ or the *SD1*^TN^ allele, which indicates that Indel_11 but not SNP10^A/G^ represents the causal variation. We conclude that the genetically determined natural variation in SD observed in our natural tomato population is largely driven by variation in the expression of *SD1*. The expression differences of *SD1* between a thick-stem accession (AC) and a thin-stem accession (TS-19) allowed us to further conclude that Indel_11 in the promoter of *SD1* was responsible for its expression. Moreover, we found that the Indel_11 was located within a putative GARE motif that could be directly targeted by GA signaling regulators that regulate *SD1* expression and subsequently SD (Qin *et al.*, 2011; Xie *et al.*, 2016). In our study, the GA_3_ treatment experiment has been proposed to explain the response of *SD1* expression to GA; however, further evidence is necessary to identify the key upstream regulatory genes.

In conclusion, our results indicate that *SD1* contributes to the genetic and molecular mechanisms that regulate stem thickening and that *SD1* also affects fruit size in tomato. We established that the key determinant of the natural variation that influences stem diameter in natural populations of tomato is Indel_11 in the promoter of the *SD1* gene. Moreover, in addition to *SDR9*, which was rigorously verified using transgenic lines, we found eight other loci that influence SD (see Supplementary Table S5). In particular, the three repeatable loci, including two significant SNPs (*P*<1.2×10^−8^) and one suggestive SNP (*P*<2.4×10^−7^), should be fully explored to help dissect the molecular basis of SD variation in tomato. These data from tomato improve our understanding of natural variation and the regulatory mechanisms of stem development in horticultural crops and might contribute to the development of breeding strategies for high yield and growth vigor.

## Supplementary data

Supplementary data are available at *JXB* online.

Dataset S1. Amino acid sequences of 33 *SD1* orthologs in plants referred to in Supplementary Fig. S2.

Fig. S1. Genome-wide association study on stem diameter in tomato using a compressed MLM model at environment 2.

Fig. S2. Characterization of *SD1* gene structure and phylogenetic analysis of *SD1* orthologs in plants.

Fig. S3. Development of an Indel_11-based codominant molecular marker.

Fig. S4. Number of cell layers of secondary phloem and secondary xylem in the young stems of *SD1*^TK^ overexpressing and *SD1* RNAi lines.

Table S1. Data for SD values detected in the GWAS population in two environments.

Table S2. Data for three fruit size related traits detected in the GWAS population.

Table S3. List of primers used in this study.

Table S4. Natural *SD1* sequence variation in tomato accessions.

Table S5. List of nine detected and suggested SNPs (including four significant SNPs) in at least one environment.

Table S6. List of the 47 SNPs significantly associated with SD in tomato.

Table S7. Genes within 200 kb of the SNP (SL2.50ch09_68226018) most highly associated with SD.

Table S8. Genotype of Indel_11 in 240 tomato accessions.

eraa144_suppl_Supplementary_Table_S1

eraa144_suppl_Supplementary_Table_S2

eraa144_suppl_Supplementary_Table_S4

eraa144_suppl_Supplementary_Table_S5

eraa144_suppl_Supplementary_Table_S8

eraa144_suppl_Supplementary_Figures_S1-S4_Tables_S3_S6_S7

eraa144_suppl_Supplementary_Dataset_S1

## Data Availability

All relevant data are within the paper and its supplementary files.
