# Supplementary material for: Tomato SD1, encoding a kinase-interacting protein, is a major locus controlling stem development
Source: J Exp Bot. 2020 Mar 19;71(12):3575–87. doi: 10.1093/jxb/eraa144 (PMC7307856; doi:10.1093/jxb/eraa144)
Supplement: eraa144_suppl_Supplementary_Figures_S1-S4_Tables_S3_S6_S7 [file eraa144_suppl_supplementary_figures_s1-s4_tables_s3_s6_s7.pdf]

Fig. S1

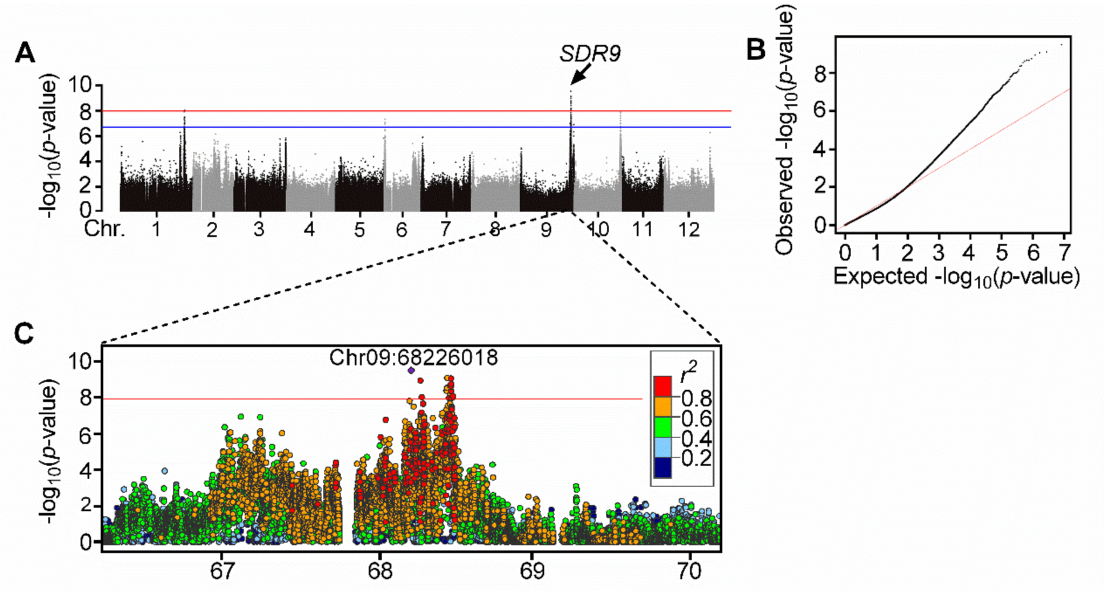

Fig. S1. Genome-wide association study on stem diameter in tomato using a compressed MLM model at environment 2. A, Manhattan plot displaying the genome-wide association signals for SD in the tomato genome (n=270). The *y*-axis indicated $-\log_{10}$ transformed *P* values. Blue and red horizontal dashed lines indicate a genome-wide suggestive (2.4×10^{-7}) and significant (1.2×10^{-8}) threshold, respectively. The arrow indicates the most significant signal from *SDR9* on chromosome 9. **B**, Quantile-quantile plot for SD in the GWAS population. **C**, The genome-wide association signal for the SD is shown on the 66.2–70.2 Mb region (x-axis) of chromosome 9. The lead SNP is indicated in purple and the colour of each plot corresponds to the r^2 value (a measure of LD) according to the legend.

Fig. S2

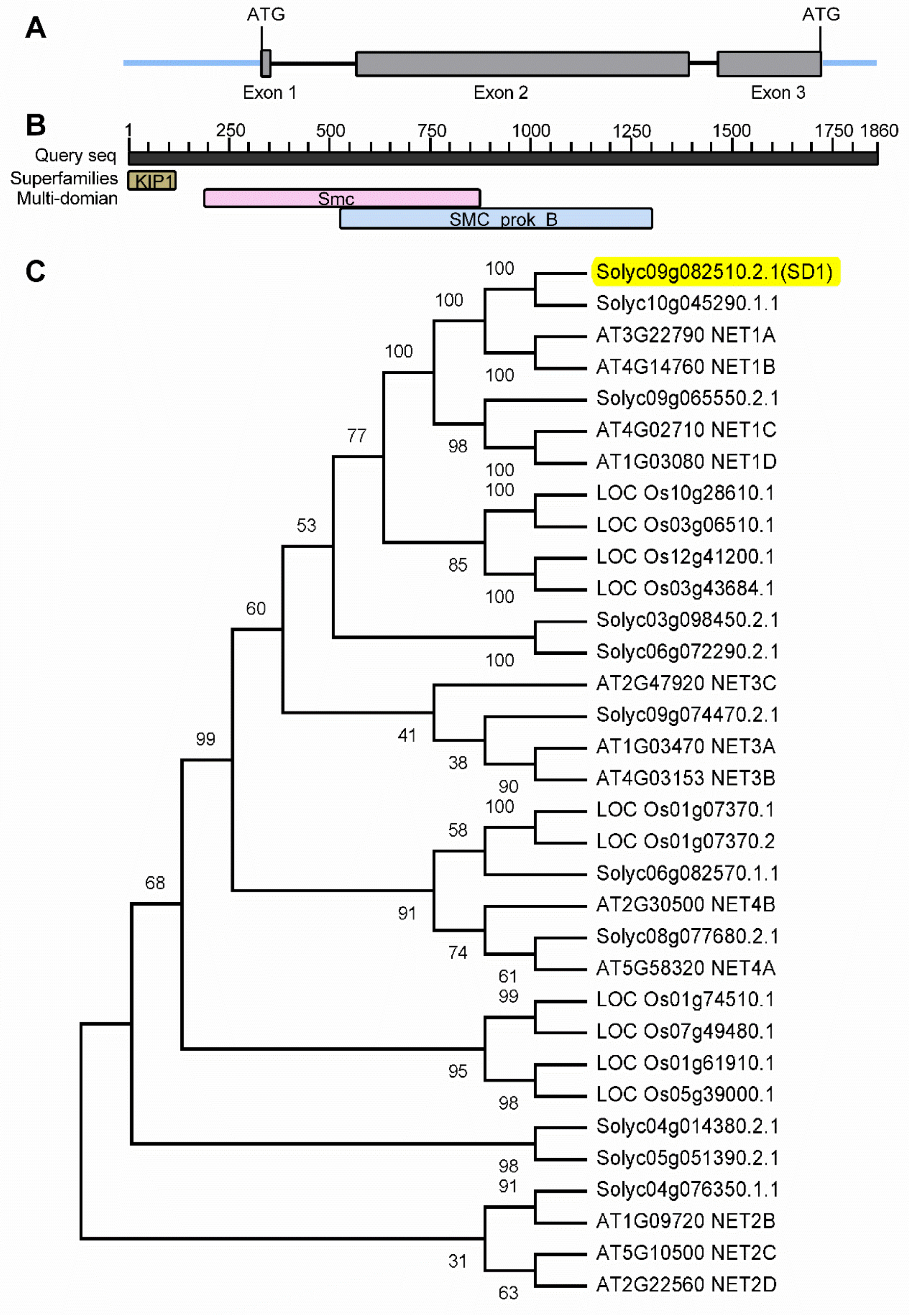

Fig. S2. Characterization of *SD1* gene structure and phylogenetic analysis of *SD1* orthologs in plants. A, Gene structure of *SD1*, including three exons (grey boxes)

and two introns (black lines). **B**, Conserved domain prediction of SD1 from NCBI. **C**, Phylogenetic tree analysis of KIP (Kinase interacting family protein) in different species. BLAST searches at NCBI were used to identify the full-length sequences of *SD1* orthologs from tomato, *Arabidopsis thaliana* and rice (see **Supplementary Data Set 1**). The neighbor-joining tree was constructed using MEGA 6 software. Numbers indicate bootstrap support based on 1000 replicates. At, *Arabidopsis thaliana*; Os, *Oryza sativa L.*; Soly, *Solanum lycopersicum*.

Fig. S3

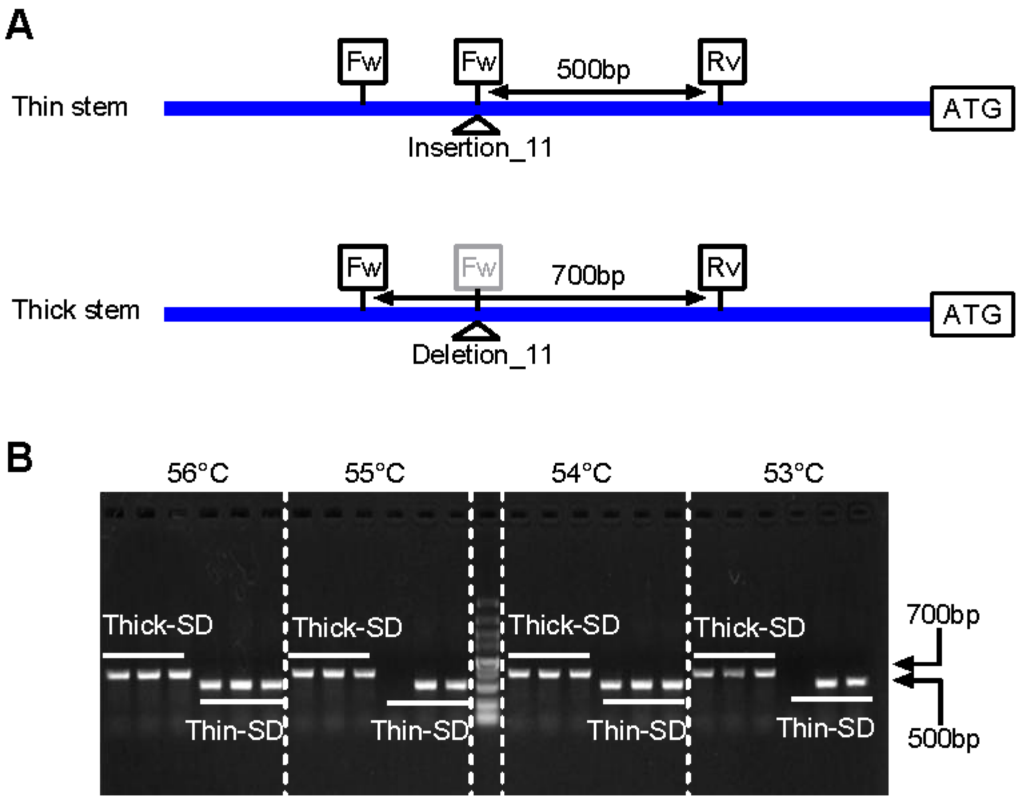

Fig. S3. Development of an indel_11-based codominant molecular marker. A, Comparison of promoter sequences between thin-stem genotypes and thick-stem genotypes. The position of the forward primer, reverse primer and indel_11 are indicated. **B**, PCR products produced with different annealing temperatures. Three thin stem accessions and three thick stem accessions were randomly selected. Marker analysis was performed using PCR amplification as described in Methods.

Fig. S4

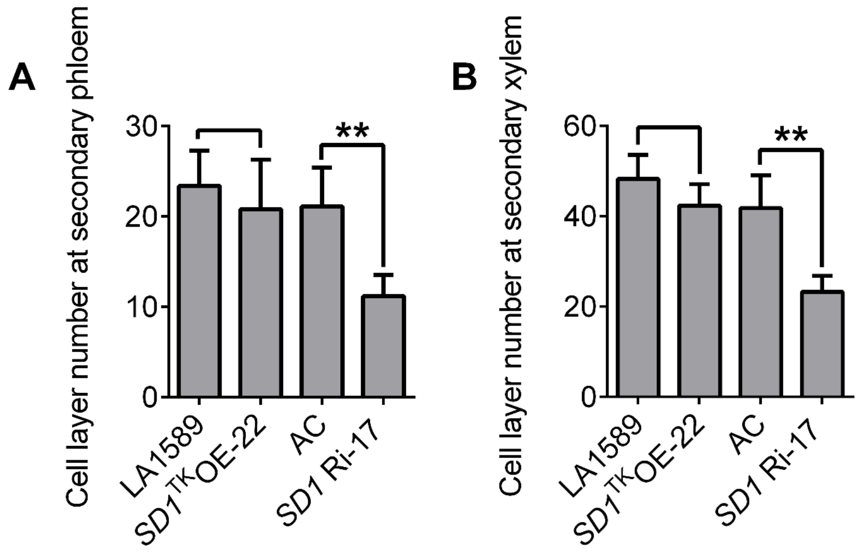

Fig. S4. Number of cell layers of secondary phloem (**A**) and secondary xylem (**B**) in the young stems of *SD1*^{TK} overexpressing and *SD1* RNAi lines. The transgenic lines were compared to appropriate wild-type controls (LA1589 and AC). All of the data in the graphs are presented as means ± SE. Asterisks indicate statistically significant differences calculated with a Student's *t* test: ***P*< 0.01.

Supplementary Table S3. The list of primers used in this study.

Name	Forward primer (5′-3′)	Reverse primer (5′-3′)
SD1 for RNAi vector	AGAGCAAGAAAGGAAATGGGGT	GAGTTGGAATGTGGCAAGGTTG
Full legth *SD1* gDNA sequencing-1	TCTTCAACATAATTTGATGGAACTA	TAGAATCCATTTCAGTAAGAT
Full legth *SD1* gDNA sequencing-2	GCTGGAGTCATGGCAACCTTGCCAC	CACCGACTGAAAGCTCACCATTAAGGC
Full legth *SD1* gDNA sequencing-3	GCCTTAATGGTGAGCTTTCAGTCGGTG	GCACATCCAGAGTCGTACACAATTCC
Full legth *SD1* gDNA sequencing-4	GGAATTGTGTACGACTCTGGATGTGC	GAGGCTTCCAGGAATGTATATG
SD1 for qPCR	ACCCGTCAGAAGCGAGTGTC	CCCGTGCTGCTCCTGGAAAA
SD1$^{InDel\_11}$ marker	AAATCTGCAACATAATTTGATGC	ACTCATGATCAAGAATGGGGTT
SD1 for overexpression vector	CATTTGGAGAGGACACGCTCGAG ATGGCAACCTTGCCACATTC	TCTCATTAAAGCAGGACTCTAGA TCATTTTGCAAATCACTTTGAAGTT
CaMV35s	ACGCACAATCCCACTATCCTTC	
SD1 for postive detect of OE transgenetic plants		GCCTTAATGGTGAGCTTTCAGTCGGTG
SD1 for postive detect of RNAi transgenetic plants		CGATACTTCTGGACTATGTGGCTCT
Actin for qPCR	GTCCTCTTCCAGCCATCCA	ACCACTGAGCACAATGTTACCG
SD1 for promoter	TGCATCCAACGCGTTGGGAGCTC ATTTGACCTTATAAACGTATTTCAGT	GCCTTCGCCATTCTAGACTCGAG GACTCCAGCAAAGAACAAAAAA
RGL1-1 for qPCR	GCTAGTGTTTTGCTTGTCAATATCA	GTCGCCACTTTCTTCATTGCT
RGL1-2 for qPCR	TGGATCTGAATCTCGTGACTGTT	TCCTCCCATTTGTGAAACTGAA
GAI for qPCR	ATTCTCTAATGGTGCTGTTTCTTCA	TTTGAGCAACATCCGCCATA

Supplementary Table S6. List of the 47 SNPs significantly associated with SD in tomato.

SNP	Chromsome	Position[a]	*P*-value
**SL2.50ch09_68226018**	9	68226018	1.74E-12
SL2.50ch09_68393289	9	68393289	2.35E-12
SL2.50ch09_68483673	9	68483673	7.64E-12
SL2.50ch09_68392386	9	68392386	1.06E-10
SL2.50ch09_68392452	9	68392452	1.54E-10
SL2.50ch09_68268899	9	68268899	2.33E-10
SL2.50ch09_68256442	9	68256442	2.72E-10
SL2.50ch09_68395704	9	68395704	4.15E-10
SL2.50ch09_68395091	9	68395091	5.35E-10
SL2.50ch09_68393178	9	68393178	5.86E-10
SL2.50ch09_68392316	9	68392316	5.92E-10
SL2.50ch09_68255001	9	68255001	1.16E-09
SL2.50ch09_68074797	9	68074797	1.25E-09
SL2.50ch09_68393185	9	68393185	1.34E-09
SL2.50ch09_68487379	9	68487379	1.70E-09
SL2.50ch09_68393281	9	68393281	1.85E-09
SL2.50ch09_68216455	9	68216455	1.93E-09
SL2.50ch09_68254247	9	68254247	2.15E-09
SL2.50ch09_68061988	9	68061988	2.16E-09
SL2.50ch09_68393216	9	68393216	2.24E-09
SL2.50ch09_68064331	9	68064331	2.56E-09
SL2.50ch09_68292672	9	68292672	2.87E-09
SL2.50ch09_68294091	9	68294091	2.97E-09
SL2.50ch09_68468703	9	68468703	3.12E-09
SL2.50ch09_68392332	9	68392332	3.71E-09
SL2.50ch09_68392488	9	68392488	3.78E-09
SL2.50ch09_68285114	9	68285114	3.83E-09
SL2.50ch09_68333314	9	68333314	4.00E-09
SL2.50ch09_68258033	9	68258033	4.71E-09
SL2.50ch09_68393011	9	68393011	4.85E-09
SL2.50ch09_68258024	9	68258024	5.85E-09
SL2.50ch09_67583603	9	67583603	6.10E-09
SL2.50ch09_68276330	9	68276330	6.18E-09
SL2.50ch09_68341636	9	68341636	6.24E-09
SL2.50ch09_68255491	9	68255491	6.29E-09
SL2.50ch09_68392618	9	68392618	6.46E-09
SL2.50ch09_68392348	9	68392348	7.17E-09
SL2.50ch09_68309561	9	68309561	8.53E-09
SL2.50ch09_68393164	9	68393164	8.99E-09
SL2.50ch09_68256179	9	68256179	9.06E-09
SL2.50ch09_68257883	9	68257883	9.40E-09
SL2.50ch09_68302113	9	68302113	9.90E-09
SL2.50ch09_68026123	9	68026123	9.97E-09
SL2.50ch09_68270379	9	68270379	1.02E-08
SL2.50ch09_68242110	9	68242110	1.09E-08
SL2.50ch09_68393153	9	68393153	1.10E-08
SL2.50ch09_68087793	9	68087793	1.15E-08

Note: The LeadSNP SL2.50ch09_68226018 is represented in bold.

[a]Position in base pairs for the SNP according to version SL2.50 of the tomato reference sequence (see URLs https://solgenomics.net/)

Supplementary Table S7. Genes within 200 kb of the SNP (SL2.50ch09_68226018) most highly associated with SD.

Gene	Start	Stop	Direction	Annotation	Distance to SNP SL2.50ch09_68226018
Solyc09g082380.2.1	68137153	68149626	Forward	ATP-dependent RNA helicase	88865
Solyc09g082390.1.1	68149811	68151412	Reverse	Pentatricopeptide repeat-containing protein	76207
Solyc09g082400.1.1	68155478	68157697	Reverse	Pentatricopeptide repeat-containing protein	70540
Solyc09g082410.1.1	68163622	68163831	Forward	Unknown Protein	62396
Solyc09g082420.1.1	68165644	68166784	Reverse	Protein RDM1	60374
Solyc09g082430.1.1	68167668	68167958	Forward	Protein RDM1	58350
Solyc09g082440.1.1	68168562	68168967	Forward	Protein RDM1	57456
Solyc09g082450.1.1	68169422	68171935	Reverse	Pentatricopeptide repeat-containing protein	56596
Solyc09g082460.2.1	68174127	68178867	Reverse	Homocysteine s-methyltransferase	51891
Solyc09g082470.2.1	68181832	68186362	Reverse	Kinase-like protein	44186
Solyc09g082480.1.1	68192090	68192574	Reverse	Protein RDM1	33928
Solyc09g082490.2.1	68200386	68204329	Reverse	Os04g0376600 protein	25632
Solyc09g082500.2.1	68217090	68223600	Forward	Tesmin/TSO1-like CXC domain containing protein	8928
**Solyc09g082510.2.1**	**68236188**	**68243577**	**Forward**	**Kinase interacting family protein KIP1-like**	**-10170**
Solyc09g082520.2.1	68244185	68246966	Reverse	40S ribosomal protein S1	-18167
Solyc09g082530.1.1	68248089	68249270	Reverse	LRR receptor-like serine/threonine-protein kinase	-22071
Solyc09g082540.2.1	68259584	68265551	Forward	Tetratricopeptide repeat protein 28	-33566
Solyc09g082550.2.1	68266303	68272143	Reverse	High affinity sulfate transporter 2	-40285
Solyc09g082560.2.1	68279897	68282072	Reverse	Calmodulin binding protein	-53879
Solyc09g082570.2.1	68295510	68298119	Forward	Neurogenic locus notch protein-like	-69492
Solyc09g082580.2.1	68322041	68325778	Forward	Os12g0604200 protein	-96023

Note: The gene closest to LeadSNP, annotated as "Kinase interacting family protein KIP1-like" is represented in bold.
