# Supplementary material for: Tomato SD1, encoding a kinase-interacting protein, is a major locus controlling stem development
Source: J Exp Bot. 2020 Mar 19;71(12):3575–87. doi: 10.1093/jxb/eraa144 (PMC7307856; doi:10.1093/jxb/eraa144)
Supplement: eraa144_suppl_Supplementary_Dataset_S1 [file eraa144_suppl_supplementary_dataset_s1.pdf]

Supplementary Data Set 1. Amino acid sequences of 33 *SD1* orthologs in plants referred to in **Fig. S2**.

> Solyc09g082510.2.1 SD1
MATLPHSNSRRKYSWWWDSHIPKNSKWLQENLTEMDSKVKAMIKLIEEDADSFARRAE
MYYKKRPELMKLVEEFYRAYRALAERYDHVSGELKQAQKTMSEAFPDQVPFLLEDSPVK
SSAHAGEPHSPEVSRGAHDFPDTGDLHQHAVGLLLSRMHAVQRSGDDKGASEWGLKQLY
EMLGAGEEMLKNSKFLEGTLKKGLSGNTEEKERSLHSQVSELSIENENLKAKVLAESERA
GQAEGEVQMLKKALAGVEVEKENTFLQYQQCLEKLSAVERDLSAAHVDSLKFNERASE
AGNEAQKLKESLIKLEAERDAALSKHKEYLERISSLEDKASQAHENTKGVNERAIKAESE
VQHLRNEICKLESEKDCCFHQYKQCLEQISELEKKLLLSQEESRLLSEKADRAESEIKKLR
DLVMELTEKKEVSVLEYKNCLEKISKLENELSRAQEDVKRLNGELSVGATKLRNAEEKCF
LLETSNQSLHSEADNLAKQITMKDQELSQKQRELEKLQSDLQNEHLRHAQIEASLLALQN
LHSQSQEEQKELALELKNGLQLLKDMETSKHSLEDELRRMKDENQSLSELKLSSTFSQEN
LENEILSLRKMKTRLEEEVAEQVELNNKLQKDISCLKEEIKDLNRSYQALVEQVKSAGLNP
ECIESSMKNLQEESSELRIISEKDRKEKEVLHKKLEDMDELLRKKAVLESSLSDVNGELQG
SQEKVRALQESCQILNGEKLTLVAEKGSLLSQLQIITDSMQKLLEKNAVLENSLFGAKIELE
GLREKSKGLEEICQLLKNEKSNLLAERGSLELQLENVERRLEYLESRFSGLEEKYSCLEKD
KKATSLEVEELRVAVGMEKQERAKLTHQSETRFLSMENHIHLLKEESKWRKKEFEEELDR
AVKAQCEIFILQKFIQDMEEKNYTLLVDCQKHVEASKLADRLITELENESLEQQVEAEVLL
DEIERLRLGIYRVFKALDNESDFVCEDRVENEQTFLHHILGNIEDLKCSLRECEDDKQQVFI
ENSVLVTLLTQLKSEAFELESVKKSVEKEFNIMAEKLVTVQKDNHELLEMNKKLGLEVSK
GSQLTAVLDAEVGSLCVKHDQLQTVYVGLKKKYSQVLEENRTLLQKITEIREEKLMVRQE
NDTLLLDTLALSNLSTVWSSFGSEKSAELKSISEDMHNLHGIISDFDKEIGILKEKLEMKET
ENLLLKESVQRLEEDLYEARESNNHLKLELSTGKEIIDKQEAGLLEAKQKLIASENLNSEL
CTTLDVLKTDRQESILTNEILEKKMLEISSTNTTQNQEIEVLREVNMNLVAEMGKLHEEIEE
QRMREEYLSSELQEKNCEFELWEAEAATFYFDLQISSVREVLLENKMNELNEVCERLEDK
NASKGLEIQRMKGKMISMEGEIGELKSQLHSYAPVIASLRDDIVSLEHNALLLMKFNLARS
QEAKCVEIEVQSGQVSSNKLTDGHSIMPKGVLDLQELRTRVKAVKKVVEGMNRPVLHQP
LHIKPGRDSTASEIESIKSRPSLDREKHEVAGRRSHQKEHEDDRNRRKAKPKSFEAKNGTL
MKDIPLDHVSDSSPERIKRAHSAAERVDDQMLELWETAEGGSLSRSVNDLKKRANHPTM
GVPIMHNQFRNLEWRGKHPPTESEVEKELGVDKLELSMNSSEANQEMNKKILKRLASDA
EKLMSLQLTVDSLRRNLEANKKAKKPKNFDFETVKEQLQEVEETVVHLVNLNSQLMKNT
EESTSYSPSSGSADSIEVMSTRQKRVSEQARKGSEKIGRLQLEVQKIQYILLKLDDEKKSKV
RSKFSRSSTGIILKNFIHIGRRNSEKKKKSPMCCFRPSSSSSSNNGSIRYRV

>Solyc10g045290.1.1
MAWIDNLLYWFKDQDFARTKPVALLFFAGVMATLLHSETKRLYSSWWDTGHIQNNSKWL
QHNLTEMDAKVKAVIKIIEEDADSFARRAEMYYKRRPELMKLIEELYRAYRALAERYDHV
IGELRQAQKTMSEAFPDQLPFLLAEDSPMRSSTQITEPHTPEILCLRASSYTHEFHQSTTGLI
PSGIHAALKIGSHNGDSNKGTSDWGLKQLLEMLGAGEEMLKSTKFLEGKLSIGLNRNTEE

KEKCLHDKVSELSNEDGNINSKILALDESEHADQAEAEVQNLKEILAVMQAEKEATVIRY
QQCMNQLYAAERELNSVQKDSVKFCEQASTAENEIQKMKESLIKLEAERDADLSKHKKC
LERISHLEVTASQALEDTKELKKRSIKAETEAQNLRNEISKLESEKDVVLHEYKLRMVNIF
DLEERLLVAQEESRMLTEIADKAEAEIKKLKIVLMELNEEKEAAGGDYKHCLDRISNLENE
LACSQEDVKRLNAEISTGAAKLKDTEDKCVVLEISKHSLYLEIDNLAKKIAMKDQELYEK
KRELEELQTDFQNLHLSHAQLEVTLQALQYLHCQSQEEQRALTMELRNSLELLKEVEECK
NSLEGELKRVTDENHSLNKLKFSLSNSIEKLENEILSLRKTEEKLEAEVAQQVGLSSNIQQD
IACLKEELKDLNGSYQALVQKVKAAGINPECVDSSIKSLQEENSNLRIICANTKCENDVLH
KKLEDIDELLKKKAVLESSLSGVTGELQGSQEKVKALQESFQILNGEKSILVAEKAALLSQ
LQIITEKMQKLLEKNAMLENSFLGAKVELESLTEKANSFEEICQLLKTRVKESEEKYACLE
KDKQAEQLQLQELRVSVKMEKQEKINFMHQSETRLIHMENHIHHLQEESKWRKKEFEEE
LDKALKSQFEIFILQKFMQDMEEKNFSLLIECQKHIETSKLSDKLITELENHILKQQVEADL
LVDEIERLRMVIYQVFKAIENDSDFASEGKVETEQTFLHYILGSVDDLKLALRMYECDKQ
QLLIENSDLLNRHAQLKSEGLELESMKKSIEEELNIVAEKLVKVQKENHCFLEMNKKLQSE
MSSSTQLNAILEVEVQTVCVKHGELQTAYFQLQTKYSQVLHQNETLLTKISEIKEEKWIVE
QENDVFLLEILVLGNFSDILKSYSSERTAELESIFEDIRKLHGATLDLEKEMDVLNGKLEMK
ETENLLLKKSIERLEEELHGVRESNDHLKLEMSTGKELQGKQEIQLMEAEQNIKVSEKLNS
ELHRALDVLKTDCLESSKLNEDLEKKIFEMLRDNTTQNKEIGSLQEANTNLVVELGKLRE
EIEEHRIRENCLSSELQEKDYEFGLWEAEAATFYFDLQISSTREGLMESKMDELSEIYGRLK
NENASKSLEIEQMKMLINLMESEIGEQKSHLHAYAPVIASLRNDVVSLEHNALLQTSLKLA
GSQEPKCVDIEVQPDESRYGNLTENQLVMTKDILDLQELRDRIKAVAEVVNKRNKPILQVS
SYNKIGRGSTETEVKESKFRYSFDLEEDEHIERRSPRNEYGEGHYRRKTKPKSFDIQKRILM
KDIPLDHVSDGSQQRIRTSGSSDVDGADDQMLELWETTEEGSPSKIMKERANHPPTESEVE
KELGVDKLTNSFDARVETNKQILYRLSSDAEKLVSLQMTVDNMRRKLDKKRKARKDKY
VDFVAAKEQLKEVELTIVQLVNLNGHLMKNTEESTHFTGSTSTYSKELLNIRGKRDLEEAR
KGSEKIGHLQLEVQKLESMLLKPGDKKKSIDRSRFYSSIALKKLIHIGKSSSEKEKNVHLC
GCFTPYNSNNISSNRYHI

>Solyc09g065550.2.1
MAALSHQDSRRMYSWWWDSHISPKNSRWLQENLTDMDVKVKGMIKLINEDADSFARRA
EMYYKKRPELMKFVEEFYRAYRALAERYDHATGVIRHAHRTMTDLGLGDDSPAGSDPQT
PELSPMLSLFDLEELQKDALGVAASNTHDLKSNGGFTDESHSGMKRKVFKQRNNLFGDQ
GRFADGRVRKGLNFSEADEKVVQTNESNSLQTRALQDSERMVESEEILKLKKALAQVEA
EKEAGLIQYQQTLEKLSHLESEVSRAREDSRGFGERASKAEVEAQTLRDALSALGAEKDA
NLKLYQKSLEMISELENTVSHAQQNSVTVDESASKAELEAQTLREDLANVAAEKDEALK
KYMQSLEMIANLENKLQCAEEDAKKLTERAETAENEIEFLKQEILKFTGEKEAAALQLQQ
CLETISTLEHKLSCAKEEAQRLNAEINNGVAKLEDAEERCLLLEKSNKSLHSELESLTLKM
GVQNQELTEKQKELGTLWTCVQEERLRFVEAETAFQTLQHLHAKAQEEMRALAPELQNR
LQVLKDLETHNQTLLGEVQKVKEENKSLGEINVSSALSMRDMQNEISSLSEAKGKLELEV
ELRMDQRNALQQEIYCLKEELNDHNKKLLSIVTQVQAVGLDPECFESSVKELQHEKSNLG
ETCERERSEKIALLEKLQVFEELLEKNSILENSLSDLSAELEAVRGSLKELEDSCQSLLQEKS
ALLNDKVTLTSELQVTIENLEEVSAKNTVLENSLSDAHVELQSLKVKSKSLEESCEVLVKE
KADLGREKENLFSQLQAAQIALHDLVGKYSGLEQRHSTLEKENELTLRAFEELRVSLDAK
NCEHESFVHTTGVRLAGMKSEIHVLQEECELRKQDFDKLLEKAIESDILNITLQTSSQDLE

GKGSSLLGEYQKLFEASTFFKTLISDLKQKNVEQKMEMTSLFDQVSILRNGIFKLLKALDI
VPNHACQDRKDQVHLDHIFHRVEASKESFDKTEEENHQRAIQMNVLVTLLEQIKLEVEAL
DAEKTIISQELNFKSEQLLALQSEAAALKEVSEELKLKIMETGHKGELLEIENCNLAKALQ
LAEDELKTVKGMMHQLNFQVVASKNLMSEKDAELQRMEQKLYLSETEKAVLHQILMNE
VAALKEGSEELKLKIREKDHRGELLVIENCDLAKALQLTEDELKTLKSMTDQLNVQVNVG
KNLLSEKDTELQGMEQKLYLTETEKAVLHQILMNEVAALKEGSEELKLIIREKDHRGELLE
IENCDLAKALHLAEDELKTLKSMMDQLSLQVNVGKNLLSEKDTELQGTEQKLYLTETEK
AVLHQILMNEVAALKEGSEELKLKIREKDHRGELLEIENCNLAKALQLAEDELKTVKSMT
DQLNLQVNVGKNLLSEKDTELQGMEQKLYLTETEKAVLHQIFKNLSRELIGSKIIMEDQEK
KILKLCADRNQLRTENMHLFEASLLLQEGLQQSRGELEKLKMQEEALHSELQKQLNETET
WKLEMDVLLGELQVSMFYHILYEQKIHELAEACQSFDVQINSKDKNIKLLKEKVLTLSTE
NEDLNTQLAAYRPAIFSLSQCISSLEKHSYLHGKPKRPDNEDTKDIVVAHTDDSTRLKDNE
NAVATDAFFDLHGLEIRVRAVEKTLVEMEQLVVKENVNMHSKLQAAMLQIEELKSESSRH
RRNSAPKSEIFEAENGILTKDIMLDRVSESSSYRNGRREQAESNNLVFDLWDTTSPTVSKA
KLDDTPNADNDIDFHKRVISVKKKCQHSTSDVLDEKYPGEGKLNISKRSTESIQEGNKRRV
LQRLDSDVQKLTNLQITVVDLKRELEITEKGKRGKAVAESDTLKGQLNEAEAAIHKLFDLT
GKLMKNMEDTFGSADMKSALESEEVGNVSRRRYSEQARGISEKIGRLQLEVQKLQFVLL
KLNDESKGNSSRIPETKRRVLLRDYLYGGVRKSNNKRKKAPFCACIQPPTQGD

>Solyc09g074470.2.1
MVEAKDKLSSHWWWLDSQKKGTLNRSPWLQSTLSELDEKTESMLRIVEQDADSFAQRA
EMYYKKRPQLINMVEDFYKTHRLLAEKYDQIKSESGTRLMTQPWMSPLSFTKYHPQKTL
MSATEISYDSYSEIFDPESELSDNMSEVEDPDLEEEEEEIQTPKSEKETMEVSSGFSVKNDE
VVKLMEEIEKLKEENRVQQELLSQKDEEKREVIRQLSLAMDLLREENIMLRKKSVATPKSS
PKKENIFEYKTLKEGFRKASSSPKKESVIELKTFKDGFWKRLFN
>Solyc03g098450.2.1
MKAKLKTEKKALSPVSSMASSNYTKRRSFSRPSWLLCTVADLDEKMNKVALKIPGKGSP
DSFAERADAYYQKRPQLMALLQELYNSYVSLADRYCQALAKNHNHRRYSSPAPPLSYNQ
NGYCDEEEYGGDIIDSDAESSLSFQPSFPPSTQDKFDIEMIVADLVIRNVDYDFVLEELNQV
ERQSNESSRKIELQKSLLDVMESERLILLNENARLGYKVATLMEENKAVSSESLFMKRKVA
ELAGCILKMREDHRVCMLSRKIEDLQGQIYGLEKRNKEYYDQLVKHEEEKTRRSKSMKV
KGEANMKYCFKVPEDVVAGITRSFSFGNLKKGSGEHKVNANAEVKKKVPKLWDRVKKF
DIFFCGPNFNTVYC

>Solyc04g014380.2.1
MLQRAASNAYSWWAASHIRTKQSKWLEQSLQDMQGRVETVIKLIEEDGDSFAKRAEMY
YKKRPELINFVEESYRAYRALAERYDHLSKELQTANNTIATICPEQIQLAMEEEDEYGAPTP
RMPKDFTQIPPNGSSNIPKAPIKDLKGFMSTTTKQRQGKKLTDDADKNDVAKSGLSKNEAI
EEIDKLQKDILALQTVKEFIRSSYKNGLERYRGIENQIMEKQQKICTLEDEFGEGQVIEDAE
ACTLMAEAALQSCQETLNHLQEKQDVYTQEARDEFNKIDDSCKKLKSFRHKYLPGQIDE
LKADRVKFPNQQVSKEIESLQDKIKDQMDASSKGSLTMSQLAEKIDELVNKVISLETEVAS
QTLLIDRLRREANELQTQVQSLEDDKAAQTGDTYNLNIRVTAIEAKLATIDNINKDVVNQ
DSSLRTHFVETRANIEHLSGKLSSVQPDEELDGTDSSPNEVTMRQDPVTQKDYPSSGEGH
KEHNSSQSNKGEFKQSTKKHVTFLQPITAGKGNVKVSAQSGTSVYETKIEEVAEKDDDLN

WQQMLLSGLEDKENILLNDYKIILKDYKEVTKRLSDMEKRDREIEFDLTLQIREFKYAITK
RDEEIHNLRRKLSLMHQGHASDQGKELKEENPSSDRSLKPDDLPQRKDNDTPIVEHDEED
IKTILVDRRASVLSPIEGEIRFSINSILDENLDFWLRFSSTFHQVQKFKTTIHDLQLEISKLKD
KEMQDKSEIRPLYKQMKEIHSELTMWLSHTLLLKDELDRKFSALCSIQDDITKALKEGVAL
DGIGLSSNEAAKFQGAVFNMKQENNKVREELEAGVRRVTTIQLDVEKTITQLDQEFGLNG
NQSQLMHTVSKSRIPLHSFIFGTKSKKQKRSVFSRIHLNRKY

>Solyc04g076350.1.1
MLHRAARNAYSWWWASHIRTKQSKWLDENLHDMEEKVEYVLKIIDEDGESFAKRAEMY
YRRRPELLNFVEDFFRSYRALAERFDHLSKDLQTANRTIATVYPERVQLAMEEEDGEQFY
AELGPPKESNEPSKSSPAAPKLSFPKVPSFAKRNAKPSRLMSKKGLIKFNVDDEIAAARPRS
GLKKSEALQEIDKLQKDILALQTEKEFIKSSYENGLAKYWEIEERVTVLQSKVSSLQDEFGI
GTMIEDDEARSLMTATALNSCQETLARLQEKQKQSVEDVIVEHQKILEVLGKFETLKGKV
VSRLPHPQVLSEVNKSSNRDSELKLLNRQVETSERKKHNEETVHKELVASSGSSMSDLAE
KVDELVDKVINLESAFSSQNAYVNRLRAETNELHSHLTKAEEEKGTLVEDTESMSKRIRQL
EEELLRVQNLNERFSFDDLSEKLNNNRQDEGVKVTLPSNKVINISGVDNAKQFQAHGAVI
PGENVVGSSNDVVSKECLKKEGLEGQEDCHEVDHQNLVESHLSSNTRYGQTGGTLGANQ
GVSGVEKAKQFQVNEATIPGEDVPGISKGVSSKEGLKKEVLQQQEGCLEVNQKNLVKSHI
SNDTRDGQTGITLEDNQGSGFTDKEMMMTTASTTGKEEKFPKADLQTEQDSFIKSTVGDS
ADVGIEKGSDKSHGHVTDHGSYTKKNDGKVEPRGTYDSANRSNFLGSAPGYDAMISTDT
KMGEEAKRRDILDNLIPFKGEAEKFFVSDLQNDHDDSTKIGCVKDVEEVKLEKGVDKSQ
QVHVTDHGSPTKKNVEQKGGNNLADRRDIFPSSINDKEEKHYKTNHQNHLNDFIQEAPPR
NSPVESESFHTSNDDLTTKRYAGKEEQARIKDAADRSDYFHIVEGKEETFYKTHDQNHIM
YCSQNLMQMKSDESNLHVSDNESAIRKDMRMEEQAGTKDIAEKSGRLQIPPEGKEENIQ
KSNHRDYQYDLPANVSISKTSNTKMEKAFHESEPAHNSDDSAAIREERTEELAVKDASHES
LSMSSPYDSDISKDIRVGQQARRTNITDKFASSTSEEGRSLSNDLLQVSPSGATLDGTSKGG
SNKDVHTMNHPRHDSVFLEDTEKSDQSEDTRASSSGSGISITTNNKGRAKVGDDHHLNM
NDSKQSDDLSRRMDFKNKIESEEQNNEFSHNFIQEKQDVDFQQSNWHNQLETHSARVHV
KDIPGPVPDDELEEWGSILDNLPSVLSDYKRDDLHKDELPDQENGKDSRRQVFRTIHNELY
TEQHESGTEDDQPNWRALFLNSLDDDREKILLEEYTSVLRNFKGVKMKLNDEEKKRRAS
HFQYVVQMKVLKNANALKDAQILSLQHRLNSLQSKDVETINFNEMPKEATFQAADMRQ
ESTAPEDRRISDFGEDSRDIKVTDVDETHSFTTVEEKVRMDIDDLLEENIELWLRFSTSFHQ
LHKFQTSVQDLQDEINKVREERKQDTGLPQSLLSGIRPIYRHLREIHTELTLWLDHNAVLK
DDLQNRLSSLSNILDVITRLSRSKDNEPVLNAYQAAKFQGEILNMKQENNKLAGELEVGIE
CIRKLQNEIQSTLSNLDEELGLKKQPARPRSKIPLRSFLFGVKLKRQRPSIFSCVNPALQKQY
SDL

>Solyc05g051390.2.1
MLQRAASNAYSWWAASHIRTKQSKWLEQSLQDMQEKVESVVKLIEEDGDSFAKRAEMY
YKKRPELINFVEESYRAYRSLAERYDHLSKELQAANNTIAAVFPEQIQLAMEEEDEYGAPK
TPKITPQIPTSSGSNVPKVPKAPIKQLKGLITSASKKLQGKKTSKQIDKSKKVPKSGLRKNE
ALDEIDKLQKDILALQTVKEFVKSSYESGLSKYKGIENQIIEKQQKIGKLEDEFGEGRVIDD
NDARTLMAEAALKTCQETLAQLQEKQERSTRDAIKEFEKIEDVSKKLKSFKHKHLGDQID
ETKKDNADKVAAKSQSLSQELSKEIESLQDKIKEQFDTSSMSSLTVTELAEKIDELVSEIVS

LETAVSAQTVLIDRVRSEGDDLQSQIHDLEDDKEPLTDDDSKQNLKISVMDMEDKLHSVQ
NLNKDVEYQNSSFQTYFTTARTSLNCLAEKLSSLKPDEEVQDEEESSVVIVKSQEEPRKQQ
VRQNASELISKTEHQQVRKEESSLKVVSDKEGEVIETTKSHSNSKHLDPTQVENEELSHED
EEKGDEPNWQELSSRSEDREKTLVEEYTTVLRNYKDVKKKLSEKEKKDRDTEFEVTLQM
RELKTAIAKRDEEINSLRGKLNALQGDNVTESKALEPSEKQAASDPSDDQSLQKSEDMAE
TEDNDNHKDQDNTMIDDHTSRSPFEEKFRLEIDAILDENLEFWLRFSSTFHQIQKFKTTVQ
DLQSEILKVREKEVEEGNNTNTDMKSEIRPIYKHMREIQNELAVWLKQTVPLKDEMKRRS
STLCRLQEEITKALKDGVEEDEIRFSSHQAAKFQGEVLNMKQENTKVKKELEAGVDHINA
LQLDVEKTVTKLEKEYGLAGGNQQQVNNSAGGAIPLRSFIFGTKPKKQRRSVFSSFQNNR
KTLWAGAPL

>Solyc06g082570.1.1
MLKMIEDSNAESFDIKYSESKKELMNLVVKCYKIYCSLAERHNDGATELLNYIPNSDLIPS
QSNVTSFKTSALPLSKFPSSKQPNHHEYKGSKQLKRGVSDKGINHHIKGKISKIPTVMYKC
KLAEFVQDSDTQSDNDYEGSDDELEAELRDMIEKLYKELEGVSNLQGETLRHGEFSNVK
KESVSSNLEIQPETSRGLYTNEPCESLEEALIQARAEKIEAETEVERLKASIVKKNNYVQEL
NKILEAVKAERDQLKVRVAMLHDDPESAKPLHQND

>Solyc06g072290.2.1
MEAKLKTEKTGNASKNSRKRSFSRPSWLLCTIADLDTKMKKLVVNIPNKYGADSFTERA
DAYYQKRPQLLALLQELYGNYLSLADRYCQTLAKNHHRQNSFPIPSFHYDHDNDDQFDK
EENNGSEIIDSDAESSLSYQPPFPSTQAKFEPDMIIADLVIRSVDCEIILHELSQVDKHCNESS
RKIELQESLLELLESERLILLNENARLGYKVGSLMEENKGLSSESLFMKRKVAELARCMLN
RRENHRVCMLSRKVEDLQSQIYGLERRNNEYYEQLLKHEEEKRGRSKMGLKGCFKVPEE
AVVGNVKKGEQQRTVGAEVGKKVPKFWNRVKKLDIFLCAPEFN

>Solyc08g077680.2.1
MESKKSHAWWWDSHINPKNCKWLQENLEQMDQNVKRMLKLIEEDADSFAKRAEMYYQ
KRPELITLVEEFYRMYRSLAERYDHVTGELRKNIPSDLQSQGSGISDVGSEPPSRLPSPDRR
PSRPKPGPRAAGFEFFLGAGGSSSDLAKEGDESSTLDSESESDDSSINNYSSTISNDDDHGL
RKKIGELEVELRDVKEKLRKQHEEVSESSVRGSHVDNDNLIARISGYEEDLRNAKEKIRLS
EEEISRLRIELQKYVSGDFVKNVHELTAGQEAKISDEGLQENAIEEEPQVLDPEGKIRTLEE
ELRSTIEKLHDSEKEVERLRDELKSNGSSVKLLHEQLGSAQKDISGWKAKLEREKREVTK
LQDRIARYKSNLSDRDQEIRGLKESISNANKALAEENLDLQCEITKLLKERAYLEDSIKEM
DLRCQSLEEDVRRAVAGKEEMEMLLKSEIEQLKLTIAERDNHIEELNRNLDALYQKYDAL
QTEKDNLNARVALLDEDLRTKDDQIDQMNNHLNQLHIEHVKLISETEGARKLVEELRSRV
KEVEREVERQKEIVLEGAEEKREAIRQLCFSLEHYRNGYQRLRQALEHKRLPVMAA

> AT3G22790 NET1A
MATVLHSESRRLYSWWWDSHIPKNSKWIQQNLSDMDSKVKAMIKLIEEDADSFARRAEM
YYKKRPELMKLVEEFYRAYRALAERYDHATVELCHAHKTMAEAFPNQVPFDMIEDSASS
SCSEPRTPEKMPPGIQPFYDSDSATSKRGLSQLTEYLGNSETEVESLKRTLVELGAEKEALN
LQYQLSLNKFSRLEKDLEVAQKDVSGLDERASKAEIETKILAEALAKLEAERDAALLRYN
ESMQKITELEESFSHAQEDVKGLTNRATKAETEVENLKQAHSRLHSEKEAGLAEYNRCLE

MISNLEKKVRDAEENAQNFSNQSAKAEDEIKALRHELVKVNEVKDGLRLRYQQCLETISK
LEREVSHAQDNAKRLSSEVLAGAAKLKTVEDQCTLLESSNETLKLEADGLTHKLAAKDQ
EIFQKQNELEKFQSLIEDEHSRYLEIEVSLKTLQSLYSQSQEEQKVITSELQSRIGMLRDLET
RNLKLEGDISSVKEENQNLSELNDSSMIFLETQKCEISSLKEIKEKLEEEVARHINQSSAFQE
EIRRLKDEIDSLNKRYQAIMEQVNLAGLDPKSLACSVRKLQDENSKLTELCNHQSDDKDA
LTEKLRELDNILRKNVCLEKLLLESNTKLDGSREKTKDLQERCESLRGEKYEFIAERANLL
SQLQIMTENMQKLLEKNSLLETSLSGANIELQCVKEKSKCFEEFFQLLKNDKAELIKERES
LISQLNAVKEKLGVLEKKFTELEGKYADLQREKQFKNLQVEELRVSLATEKQERASYERS
TDTRLADLQNNVSFLREECRSRKKEFEEELDRAVNAQVEIFILQKFIEDLEQKNFSLLIECQ
KYAEASSFSEKLIAELESENLEQQMEAEFLVHEIDNFRGAICQVFKALQVEADCKTADQKI
AKERIPVSRVLGEINELKCSLSSAEYETQRLVIENSVLLSLLGQFQSDGMKLESEKRDVEKD
LETIVHHYGMLKKDRLELLEMNRQLKSELIDREQRELELKAELQTEHLKFENLHESYMAL
HQDYSDALGKNKSLHLKFSELKGEICILEEENGAILEEAIALNNVSVVYQSLGSEKAEQAE
AFAKNLNSLQNINSGLKQKVETLEEILKGKEVDSQELNSKLEKLQESLEEANELNDLLEHQ
ILVKEETLRQKAIELLEAEEMLKATHNANAELCEAVEELRKDCKESRKLKGNLEKRNSEL
CDLAGRQDEEIKILSNLKENLESEVKLLHKEIQEHRVREEFLSSELQEKSNEFGLWDAEATS
FYFDLQISAVREVLLENKVQELTGVCENLKDEAVTKTTEINQIKETVGFLEFEVSELKTQLS
AYDPVVASLAEDVRSLEQNALSLMKLPVPAGRRREGVQNDEHQEAAVSQEPVGHCSTNL
DNGIVLLQDMKTRIKTIKQAVAEEKKRRGKLRRRSSSHRSKDRKLFEEIELEDQFSGEIRQP
RSPAMTESKNGSLMKDIPLDQVADTTSYGRSRRTSRGSSDQMLELWEEAAEPESSIKFLIN
NKNSKKPLIPRLHRRSRNPSVESQSEKMVGVVDKLELSRSTEDNAKILERLLSDSRRLASL
RISLRDLKSKLEINEKPGKFTNPDFARVRKQMKEMEEAIFQLANTNEILSNEIEETGDVRDI
YRKVVMEKSRIGSEKIEQMQQEMQNIERTVLKLEEGATKSKGRRKFSESRTVILLRDIIHK
GGKRTARKKKNRFCGCMRSSGNEE

>AT4G14760 NET1B
MASLSQSESGRLYSWWWDSHIPKNSKWIQDNLADMDSKVKTMIKLIEADADSFARRAD
MYFKKRPELMKLVEELYRAYRALAERYDHTTVELRRAHKVMVEAFPNQMSFDMIEDSAS
SSSEPRTEADTEALQKDGTKSKRSFSQMNKLDGTSDSHEADSEVETLKRTLLELQTEKEAL
NLQYQLILSKVSRFEKELNDAQKDVKGFDERACKADIEIKILKESLAKLEVERDTGLLQYS
QAIERIADLEASISHGQEYAKGLTNRVSEAEREAMSLKKELSRLQSEKEAGLLRYNKSLELI
SSLEKTIRDAEESVRVFRDQSEQAETEIKALKQELLKLNEVNEDLNVRYQQCLETISKLER
EVSHAQDNAKRLSSEVLAGAAKIKTVEEQCALLESFNQTMKVEAENLAHKMSAKDQELS
QKQNEIEKLQAVMQEEQLRFSELGASLRNLESLHSQSQEEQKVLTSELHSRIQMLRELEMR
NSKLEGDISSKEENRNLSEINDTSISLEIQKNEISCLKKMKEKLEEEVAKQMNQSSALQVEI
HCVKGNIDSMNRRYQKLIDQVSLTGFDPESLSYSVKKLQDENSKLVELCTNQRDENNAVT
GKLCEMDSILKRNADLEKLLLESNTKLDGSREKAKDLIERCESLRGEKSELAAERANLVS
QLQIMTANMQTLLEKNSVLEKSLSCANIELESLRDKSKCFDDFFQFLKNDKSELMKERESL
VSQLCKVEEKLGVLEKKYTELEVRYTDLQRDNKLKSHQVEELQVSLAAEKQESANYKRS
TESRLADLQKNVSFLREECRSRKREYEDELDRVVNKQVEIFILQKLIEDLEQKNFSLLIECQ
KHVEASEFSEKLIAELESENLEQQMEAEIFLDEIDSLRGAIYQVIKALQVEADCKTEQKITK
DQISVSRALGEIDSLKGSLSSAEYEMHRLVVENSVLLSLLGQFQSDGLVLESEKNILEKDLK
TKIHQCGMLEKDKQDLQEANRLLKSKLIKREQQEQKLRAELKFENLKFESLHDSYMVLQ
QDYSYTLNDNKTLLLKFSEFKDGMHVVEEENDAILQEAVALSNTCVVYRSFGSEMAEEV

EDFVETVSSLREISTGLKRKVETLEKKLEGKEKESQGLNKMLENLQEGLEEDNFLTGLLEH
QVSNVDEILEHREMEILEAEHMLKATNNENEELHKEVEELRKDYEDSRRMRANLEWQISE
LSDVAGRQEEEIRKLNALNENLESEVQFLNKEIQRQQVREEYLSLELQEKSNEIGLWDSAA
TSFYFDLQVSAIRELILENKVNELSGVCENLNDEVVTKTTKIKQMKETVGFLESQVTELKS
QLSAYDPVIASLAGDVKALEKSTHALTKFPATAYQQRVGNNLEESGSTTSPCNGIVILKEIN
PSIKTIEQAFVKEKGRLSRQITRSTSQKRRDRRKIENIQPDDQVTGESRQPRLRPEMTEVKN
ELLMKDNPRDQVTDSLTYGRSQGTSHGSNDMFEFWDESAESETSVNFLINSNKPQRSLNS
NLRHQSRNPSIESDKAVGVVDKLELSRNIEDKAKILERLLSDSRRLSSLRISLTDLKRKLEM
NEKQRRFSNADLVIVKRQLKEMEEAVSQLENTNEILSKEIEETGDARDIYRKVVVEKSRSG
SEKIEQLQNKMQNIEQTVLKLEDGTKSKGRKMFSETRTVILLRDIIHKGGKRSARKKKNRF
CGCIRSSTKEE

>AT4G02710 NET1C
MEIAAKSNSKRMYSWWWDSHNTPKNSKWLQDNLADMDSNVKQMIKVLEEDADSFARR
AEMYYRKRPELMKLVEEFYRAYRALAERYNHATGVIHKAHETIAEAFPNQVPLIFGDESH
GGALTNDVDPQTPDMPPPFRARGNPDEFQQDALGFSLSHVHDVKRNIDFSEEPLFVSNGK
ARKGLNFNDHGDGKGRNGLKDHILSESERASKAEAEVVALKDSLSKMQAEKQASLALFE
KNLERLSNLESEVSRAQADSRGINDRAASAEAEIQTLRETLYKLESEKESSFLQYHKCLQK
IADLEDGLSVAHKEAGERASKAETETLALKRSLAKAETDKETALIQYRQCLNTISNLEERL
RKAEEDARLINERAEKAGVEVENLKQTVSKLIKDKEASELQFQQCLNIIASLKVKLHHAQ
EETQSLSHEIEDGVAKLKFSEEKCLLLERSNQNLHSELDSLLEKLGNQSQKLTEKQTELVK
LWSCVQAEHLHFQEAETAFQTLQQLHSQSQEELNNLAVELQTVSQIMKDMEMRNNELHE
ELEQAKVENKGLNDLNFTMEKLVQKNLMLEKSISYLNSELESFRRKLKTFEEACQSLSEE
KSCLISENQHNVIENTVLIEWLRQLRLEAVGIATEKTDLEGKAKTIGDKLTDAETENLQLK
RNLLSIRSEKHHLEDEITNVKDQLHEKEKEFEEIKMEKEKLIQEVFKERKQVELWESQAAT
FFCDKQISVVHETLIEATTRELAEACKNLESKSASRDADIEKLKRSQTIVLLNESIKSLEDY
VFTHRESAGEVSKGADLMDEFLKLEGMCLRIKAIAEAIMEKEKFLMLENTNTYSMLEASL
KQIKELKTGGGRSMRKQDGGSGRMRKQSHETEMVMKDIVLDQTSDGSSYEIVSKKGNSE
LDHLGFVELKPVKTHKTETKALSEESLIVEKVEIFDGFMDPNREVNKRRVLERLDSDLQK
LENLQITVEDLKSKVETVEKEKTKVGENEYKTIKGQLEEGEEAIEKLFTVNRKLTTKAESE
KDIDRRRRIFEHARRGTEKIGRLQSEIQRIQFLLMKLEGEREHRLRSKISDTKVLLRDYIYG
RTRSVSMKKRTKKRSVFCGCVQQPESP

> AT1G03080 NET1D
MTAVVNGNSKRYSWWWDSHISPKNSKWLQENLTDMDSKVKQMIKVIEEDADSFARRAE
MYYKKRPELMKLVEEFYRAYRALAERYDHATGVIRHAQQTMAEAFPNQDPMMFGEESP
LGSSTDGFDPQTPDSYPPIRAPVYPDDLRKGAFGISSSHLSTVKRNIAFMEDPQSVSSGKGF
KTAKARKGLNFNNVDGKEINAKVLSESERASKAEAEIVALKDALSKVQAEKEASLAQFD
QNLEKLSNLESEVSRAQEDSRVLIERATRAEAEVETLRESLSKVEVEKESSLLQYQQCLQN
IADLEDRISLAQKEAGEVDERANRAEAETLALKQSLVSSETDKEAALVQYQQCLKTISNLE
ERLHKAEEDSRLTNQRAENAEGEVESLKQKVSKLIEENEAYELQYQQCLDTIADLKLKLF
HAQEETQRLSREIEDGVAKLKFAEEKCVVLERSNQNLHSELDGLLEKLGNQSHELTEKQK
ELGRLWTCVQEENLRFMEAETAFQTLQQLHSQSQEELSTLALELQNRSQILKDMEARNNG
LQEEVQEAKDQSKSLNELNLSSAASIKSLQEEVSKLRETIQKLEAEVELRVDQRNALQQEI

YCLKEELSQIGKKHQSMVEQVELVGLHPESFGSSVKELQEENSKLKEIRERESIEKTALIEK
LEMMEKLVQKNLLLENSISDLNAELETIRGKLKTLEEASMSLAEEKSGLHSEKDMLISRLQ
SATENSKKLSEENMVLENSLFNANVELEELKSKLKSLEESCHLLNDDKTTLTSERESLLSHI
DTMRKRIEDLEKEHAELKVKVLELATERESSLQKIEELGVSLNAKDCEYASFVQFSESRM
NGMESTIHHLQDENQCRVREYQVELDRAHDAHIEIIVLQKCLQDWLEKSSSLIAENQDIKE
ASKLLEKLVSELEEENIGKQVQIDSSINCIKILRTGIYQVLMKLEIIPGIGSGDENSRDQRNM
HDILNRLEDMQTMLLSIRDENQHSAIENLVLIEFLRQLKSEAVGIETEKKILEEELESQCQQL
SFSRDETQKLIFVNGELTTKVNQGVNREKVLMVEIEDFHRQVLQLRDDYTILQGDNNKTL
DEKAYLTKSTLQLEEEKCKLEDDISLLLSETIYQSNLIILLEDVILEKLSGAMKLNEDLDRLS
IVKCKLEEEVRELGDKLKSADIANFQLQVVLEKSNAELLSARSANVHLEHEIANVKVQKE
KELLEAMLMISIMQNEKSELSKAVEGLECRYKEAKAIEEDRDKQVLRLRGDYDEQVKKN
SHSNEANLKLEADLMNLLMELEEIKVEKENLNQELFTERNEIELWESQSATLFGELQISAV
HETLLEGLTNELVEACKNLESRSTLKDREIEQLKGRVNNLEDANKGQNDLMCKYAQAIFL
LKESIQSLEKHAMLHEFENGPATETASLVDNSDGFLEIQELHLRIKAIEEAITKKLAMEELK
TSSARRSRRRNGSLRKQNHEIYSEETEMITKDIVLDQVSDCSSYGISTRDILKIEDDHSLEA
KSQNPPKGKSLSEESLVVDKLEISDRFTDPNKDANKRKVLERLNSDLQKLSNLHVAVEDL
KIKVETEEKDEKGKENEYETIKGQINEAEEALEKLLSINRKLVTKVQNGFERSDGSKSSMD
LDENESSRRRRISEQARRGSEKIGRLQLEIQRLQFLLLKLEGDREDRAKAKISDSKTRILLR
DYIYSGVRGERRKRIKKRFAFCGCVQPPPSP

> AT1G09720 NET2B
MLQRAASNAYSWWWASHIRTKQSKWLEHNLQDMEEKVKYTLKIIDGDGDSFAKRAEMY
YRKRPEIVNFVEEAFRSYRALAERYDHLSTELQSANHMIATAFPEHVPFPLVDDDDDDDD
DNPKKPPKHLHLIPSGTNIPQVPEVPKKEFKSQSLMVLSRKEPGVLQSSETSSALVSSGLSR
EEALEEIDKIHKGILVLQTEKEFVRSSYEQSYDRYWNLENEVEEMQKRVCSLQDEFGVGG
EIEDGEARTLVATAALSSCKETIAKLEETQKRFSEDAGIEKERIDTATERCEALKKKFEIKVE
EQAKKAFHGQESSYESVKESRQIDLNENLSNVDFAEKIDELVEKVVSLETTALSHTALLKT
LRSETNELQDHIRDVEKDKACLVSDSMDMKKRITVLEDELRKVKNLFQRVEDQNKNLHK
HLTEANSTAKDLSGKLQEVKMDEDVEGDGLNPEDIQEEDTVEDSDSISNEREIKNAEEIKE
AMVIKQSRDQESMQEEKSETRDSCGGLSETESTCFGTEAEDEERRNWRQLLPADGMEDR
EKVLLDEYSSVLRDYREVKRKLSEVEKKNRDGFFELALQLRELKNAVSCEDVDFHFLHQ
KPELPGQGFPHPVERNRAESVSISHSSNSSFSMPPLPQRGDLKRASEQEKEDGFKVKFAGIS
DSLRKKIPTVEEKVRGDIDAVLEENIEFWLRFSTSVHQIQKYHTSVQDLKAELSKIESKQQ
GNAGSSSNTALASEAKPIYRHLREIRTELQLWLENSAILRDELEGRYATLCNIKDEVSRVTS
QSGATEVSNTEIRGYQAAKFHGEILNMKQENKRVFNELQAGLDRARALRAEVERVVCKL
EENLGILDGTATRSLSKRMPSSAGKPRIPLRSFLFGVKLKKYKQQPKQTSTIFSCVSPSPALN
KQCSYIIPPAKLPEYVKRS

> AT5G10500 NET2C
MLRRAASNAYSWWWASHVRTKQSKWLEENLQDIEEKVEYALKLLEDEGDSFAKRAEMY
YKRRPELISFVEESFKAYRALAERYDHISKELQNANTTIASVFPDQVPEFAMNEDDDDDAP
VSPRHHKNKTSNKNVPKVPDLPIKDPEAAKKMFMSRKAIQEQNASSVVNKSGLSKTEAV
EEIDKLQKEILVLQTEKEFVKTSYENGLAKYWEIEKCIMEKQGKVSSLQDEFDEGAVVIED
KEAQILMSTTALKSCQEKLEELRDKQEQNVKEVDVSRKQISESTEEFGNLSDALLGDGKG

NHEIYSEKEKLESLGEKVNDEFDDSEAKSCLTIPDVADKIDELVNDVINLENLFSSQAALIH
RLREEIDDLKAQIRALQKENNSSQTDDNMDMGKKLKEMEEKVNGVKDIDQEVEEKSDNI
DKHLTRAHMKLSFLSKRLKSLTQEGEDEELKATNVPIQDIGSLTDTKFPEENIDDTVVSEN
ALDIKSASEVVFAEKDLSDEVNQEEAIETKTKEASLSDLEKHISSPKSDIITTQESSDELFLQ
KLLAHGIEGREKHLLTEYTKVLRNYKEVKKLLHETETKLKNVNTLKDEGKDQQRGQLFM
LICREDNNATNAITGQKQRMSPNEEQLGARVDALLSENLNLLVRFSNSFGKIQQFDTGIKD
LHGEMLKIIKQKNQDGGKNTLRSNVRPIYKHLSEIRTEMTVWLEKSLLLKEEINIRASTLS
DIHNEITEALKTDSEDSEIKFTIYQGAKFEGEVSNMKKENNRIAEELQTGLDQVTKLMKDA
DTTLEKLSEEFSLSESNTQSSQDRSRIPLRSFIFDRKPKKQRLSLFSCIQPSLSKMKKPAGS

>AT2G22560 NET2D
MLQRAASNAYSWWWASHIRTKQSKWLEQNLQDIEEKVQYVLKLLQEDGDSFAKRAEMY
YKKRPELISFVEESYRAYRALAERYDHISTELQNANTTIASVFPDQVPNFAMDDDIDMSKF
AKRSNISGANVPNVPKLPVKDLKSAVRVATKKLQPRKSMKYTGGSTNVVVKSSGLSKPEA
MGEIDKLQKEILALQTEKEFVKSSYEIGLSKYWEFEKGIKEKQERICGLQDEFGESVAIEDE
EARRLMTETAIKSCQEKLVELQEKQEKSYEEAREEHVKIKESKEKLRSMASQFLGDESVFA
KDDGDEVRRTAELDHEIKEMSRKKKELESVKEKIREHFESGANSSLNGTDMAEKVDELVN
KVISLESAVSSQTALIQRLRNETNGLQTQISTLETDKALLADDKSDLRNKLKEMEEKLKAL
QDLDRNVLDKSSNLQTHFDDACHNLDNLSGGNLHEVKPESESDNLAISIEPQKDLEGEKR
TLDISEEIKEHQKETGEEKKEAPVKSVKFEQTRNATIAEDSTIPSTNPDTVLESTEKVDSDL
EKQDASDKTDSVLDNVLENQAASDQTDSVLDSVLEKQGESDKIDSVPSNVSEKESDISFN
GEQQEDQKEKEGEPDWKEMFMKGMENREKHLLTEYTTILRNFKDMKKTLDETKTKMKT
ENATKDDEIKLLREKMSLLQKGLGDSNDLMENQLSNDDYSIGFMAAENQNMSLVEEQFR
LNIDELLEENLDFWLRFSTAFGQIQSYDTSIEDLQAEISKLEQRRKQDGSSTAKYALRSDVR
PLYVHLREINTDLGLWLEKGAALKEELKSRFESLCNIQDEITKALKSSAEDDDFRFTSYQA
AKFQGEVLNMKQENNKVADELQAGLDHITTLQLEVDKTLGKLIDEFALSGSKNKSDLDL
QHSDSRSRVPLRSFIFGSKQKRAKPSIFSCMHPSLYRKMKTST

>AT1G03470 NET3A
MVMDSSKWWWIGNHNTTNFSPWLHSTLSELDEKTKEMLRVIDEDADSFAARAEMYYKK
RPELIAMVEEFYRSHRSLAERYDLLRPSSVHKHGSDSESHEKSSTCDESSWSEACETHEEY
AESEIDNGESKWVDESEIDGIVEEIEPSEVVYSEGNGNSEMMKIEIERLREENKVYSEMVR
EKDEEKREAIRQMSVAIQMLKEENSELKKRVTNTVVARRNKEGGDSQRKQQMWKPFEFK
KIKLEGLWGKGFGNWALPNTDSTSKELMTL

> AT4G03153 NET3B
MGETSKWWWIGANHNTSNSSPWLNSTLSELDSKTKEMLSVIDEVEDEGDSLMKRAKINY
ENKPKLIELLEELYRSHRSLAQKHDLLIKTSSLNSDSHNSSSCDEIRSEVCEETESSDVEAET
EKDQIVEFDDGDETMKEELEILREENRVYKEKKEVVTRLLANLVRVCFCFQFNWEIFSYH
LLRFCLLFSHDPIGGWVICTKRFRYYFLIMFSF

> AT2G47920 NET3C
MVREEEKSRWWWFESHKSSKHSQWLQSTLAEIDAKTKAMLKLLDGNADSFAQRAETYY
KKRPELISFVEDFYRAHRSLAVNFDHLKSSDHYGSRSAKVPQQSMESVCDSNSHFEDADS

EIEDPLQDDASAADCKEDETWQLEQERLKLIEETDALRKQLLDKDEEKREVIRQLSLTLET
LKDENLSLKRRLAHHSLKQRTVLEFKPLNKFFGKLFYIMCDGNKVL

>AT5G58320 NET4A
MDYDLLRSKKSIKRVESTKSNPWWWDSHIGLKNSKWLENNLDEMDRSVKRMVKLIEED
ADSFAKKAEMYYQSRPELIALVDEFHRMYRALAERYENITGELRKGSPLELQSQGSGLSDI
SASDLSALWTSNEVNRLGRPPSGRRAPGFEYFLGNGGLPSDLYHKDGDDSASITDSELESD
DSSVTNYPGYVSIGSDFQSLSKRIMDLEIELREAKERLRMQLEGNTESLLPRVKSETKFVDF
PAKLAACEQELKDVNEKLQNSEDQIYILKSQLARYLPSGLDDEQSEGAASTQELDIETLSE
ELRITSLRLREAEKQNGIMRKEVEKSKSDDAKLKSLQDMLESAQKEAAAWKSKASADKR
EVVKLLDRISMLKSSLAGRDHEIRDLKTALSDAEEKIFPEKAQVKADIAKLLEEKIHRDDQ
FKELEANVRYLEDERRKVNNEKIEEEEKLKSEIEVLTLEKVEKGRCIETLSRKVSELESEISR
LGSEIKARDDRTMEMEKEVEKQRRELEEVAEEKREVIRQLCFSLDYSRDEYKRLRIAFSGH
PPTRPSSILAS

> AT2G30500 NET4B
MASSTAQSKKQFKRSMTKKSHSWWWDSHNCPKNSKWLAENLEKMDDRVNHMLKLIEE
DADSFAKKAQMYFQKRPELIQLVEEFYRMYRALAERYDQASGELQKNHTSEIQSQSSLEIS
SPTKEKLSRRQSSHKEEEDSSSLTDSGSDSDHSSANDEDGDEALIRRMAELELELQETKQK
LLLQQESVDGDNNVDLLHKITTYEGELKEANEKMRMHEDEIANLKNQLQSFMSFDTEDH
LGAEQKSVDLDKEDTKEDAVATKVLALEEELSIAKEKLQHFEKETYSLKNELEIGKAAEE
KLKSLQHELELAQRDADTYINKLNAEKKEVLKLQERLAMVKTSLQDRDNEIRALKTAVS
DAEQKIFPEKAQIKGEMSKMLEERSQLGEQLRELESHIRLIKEEKAETEEKLRGGTEKISG
MRDESNVLREEIGKREEKIKETEKHMEELHMEQVRLRRRSSELTEEVERTRVSASEMAEQ
KREAIRQLCMSLDHYRDGYDRLWRVVAGHKSKRVVVLST

> LOC_Os10g28610.1
MAALVGHDARQYSWWWVSHISPKNSKWLQENLNDMDSKVKAMIKLLNEDADSFARRA
EMYYKKRPELMKLVEEFYRAYRALAERYDQATGALRQAHKSISEAFPNQMPPMSDESPA
SSGQEVEPHTPDLPTFTRLPFDLDDLQKDGVGVSPQQFTSKRNGTHPEEASALPNRKGFD
VKVRKGLSFGSPEVKGSDAISNEMVNLQQEISRLLAESNSMKQQILSESERANKAENEIQV
LKDTILKLNSDKDTSLLQYNQSTERLSTLESELSKAQDDLKKLTDEMATEVQKLSSAEAR
NSEIQSELEALDQKVKMQQEELEQKQKELKSFNLTFQEEQDKRMQAESALLSEGKELAQ
CQEEVQRLTKEIQMANEKLNELKQTKVNLENAVSELKKEVENLTEQNRSSELLIQELRDEI
NSLKDSKNELQNEIQSLRSTISQLNTEKDATLFQHQQSVERVSDLESQLLKLQPELEEIEQK
VQMLMQDLEQKRQEADSAHAQLQDECNRHTQTEADLHRFKNLHSQLEEEVIKLTENLD
RSTKELEELENAKLDLENTSRELKSTILDLNSEKDAVLLQQQQSLAKISELELQLSKTQLEL
KNSEQKMQLLELEITQKSESMDSLTLSLKDETEKRVQAETSLMSMESMYSQSQEEVNRLH
LEIEKLNFKLNELENLSSELNSTILLLNAEKDATDLKNQQSLVRISDLESELSKLQAQLEKIE
GKVQMLEQELKHKKEEVDSLQISIQDEAHKRSEGEAALLAMTNLNSESQEEVNRLTLETK
KLKVKLSEVENSNTDLENIVAKHTQDIHVLREKNVSTELMIKELHHELDALKELNVKLES
EMGLHIGEKEALQRDFACQKEEKQNLEGIHHSLAEEMSTLKSRSAANQKLIEDLQIMNLK
LKEVCAKNEVEKALLSEKVQEVEKLSEEFSLMENSLSDANAEMDSLREKIKVLETSEGSL
KDVISSHVSEKAILTSDLETLGKSYADISEKNSNLDILISDMKAEIENLRTKLTDSEETCQAH

LANNSALSDEKNNVFSQLESVTVVMKALESKHADLEDKSSSLSREMNLAYDQVRELQDQ
LRVKDEEYEAFVKSHQTQVNDFEEQISSLQKKSYYMNELLEQEQENHMSASINVVILENC
LADLKDKNVDLFNECQKFAEANHAAEMLISQMKDEARYHQDERKFLLIHTEKLREGISQ
HMKILNICKDLGPANIAEDKIILQTVSDEASNIMKLKEQSEDANRLMYTELTVLATVMLQV
GLELRDLNLQKRALEKELETRAAEFITLQNNNVQMLEWNEQLKQELQQGCEREEVLKAE
ILVLQEKLSCSRESYQTSQNEIVSLTEKNETLCKEYQSLIENYNALEDENGTLLSECMRLEH
LSLFLRGHNNEVATALGSLTDEMALLSVGKDELDCEVQELSRRGMMLESENNNLKEYFIY
LIEILSAQLALSEFDLNINKSICQELASELESCMAQLSQKDDELLEAEDKVHLLQGKNREL
CGVVGSLQVAIEGAKIVKEELEKKITTLTEEGNTKDGEISLLRQANERLQVEADILKDKED
SLTSSHELLSKEVEQHEGEFVVLMDDAISSSVNAAVYEEKALELMTENTELKANLSTHVA
LIASLSDHVNELEENTLSLSKPYSTESKKEDAEVPFMQERNHGPESHPLPEGTPELQRLIAR
MGALQVAIRNAKDLHDQESTKSAATLAAAHRDIQELKARGGSQMEAREIYSDNEKLNNV
EGSKGKQVQMMKDIELDQISTCPPYGTGAALYPLKNGANAGMDDEMLQLWEAAERSCK
NQTSKSSSAEHDIEAVEEVKSEYPSSELARGRDLGINKLEVSTSSVEPHEQWSNNVLEKLS
SDAQRLQSIQVSIKELKRKMGSPSNGKSPMNSEYNTVSTQLLDTEGCVLEQINYNNKLTK
RVENYPALSDSMNAEQEGYPSRRKISGQVQKGSENVGRLELELQKIQYVLLKLEEEHEYR
RLKVSDKRTRVLLRDYLYGRKEKRGGAQKKKKRAPFCGCVQSRTET

>LOC_Os03g06510.1
MEILGPLPMAESSRQAAIDGCTWVNRVATSSAANCSSTTTTTVSTCPCCCNGGGPCEPFSA
RYTLHAADSGDHRERQMGGEMGSCIACCLALPPLATACPPLVTATLRASAMEYHEWEVN
CPQLEGKFGVLFRLLALRFAWKHDIFCCCLNAYFEEVETSFVVTYDLPRCCSILSTKDIETD
RHIVPLWQLTCSFVPMASLVRHDSNPTQYSWWWVSHISPKNSKWLQENVTDMDVMVKA
MIKLINEDADSFARRAEMYYKKRPELMNLVEEFYRAYRALAERYDQATGALRQAHRTISE
AFPNQMPSMSEDSPSSGQDVEPRTPEVLMPTRAPFDLDDLQDAAGVSPHLLTVKRNGTQP
DDIGFSSSRKGLKQFSDLFAGSDSSQRVNFSDGKVRKGLNFESPDVKGKKDDSNDIMNLQ
HEVSKLLTERQSLKEQISSESQRANKAESEIHSLKDTISCLISEKDTTLLQYNESTRRLSVLE
CEISKAHMELKKLSDDMAMEVDKLKCAESQNSAMQSELETLDQKVRVQEQELEQSRKEI
ESFHLSLQDEMAKRKQAEDALCSLEKQYAQSQKEINRLTLDMEMANDRLNDFNLVRLNL
ENTVCELKKEVTSLEVKIQILVQELEQKREEADVMHAQLQDEHSNHMHKEAALHALENL
HSQSQEDFNLVKLNLENTVGELKKEVTSLELKIQIQAQELEQKREEADTVHAQLQDERSN
HMQKEAALHALENLHSQSQEDFNLVKLNLENTVCELKKEVTSLERKIQIQVQELEQKREE
ADAMHAQLQDERSNHMQKEAALRALENLHSQSQEEVKQMARDVEHSNKKLSDLENNN
LKLHDLSQGLKKTVAELNSMKDSALLQQQKSSEKVSYLEAQVLVVRSEMEKMVQKTQM
LDQELEHKNKEISELQNSLQEQVQKCILAETSLLRLEDLHTNSQKEAKTLAHDLERLSEQL
TEVENDRLDLQNISRELKNTISEINSEKDLMLLQQQHSLERQSYLEAQLLDALSEVEENKK
EAQLLEENLAHKNDELNDLQNNLEEEGHKRMHAEAALSMVENLHSKSQEEVGKLVMDL
DKLENELSELQGRNSRMEELSYELQNTISLLNSEKDAALLQQQLSSERACDLMSQLSKIQL
ELEKAEEKMQTMEQKLADKNEMVDFLQLSLQDEGKKRVEVETALISSGNLYSQSQEDVN
RLTLEIERLNEMLNDMENKSSEYKSTILLLNSEKDMSVIQYKQSSLRIYELESKLSGVQEEL
DNAEQKVQMLDKELKEKREVVETMQASLQDEAQKRMKGEATLLTMTNLHTQSQEEVN
RLTPEIERLNRKLNEVENVSCELKNTILLLNSERDTTVLQHKQALVRVSDLESELSDVQAEL
VNAEKNVQILDKELKQKREEVDSLQASLNEEAQKRIEGEVALLAMENLHSQSQEEVRGL
VLKIETLHGKLNEMENSNRDLKNMICKHSEEIHVLGEQNLSAELTIKGLHDQLEKFTEMNI

GLQNEVGIHVGEKEVLQQDLARQKEDKDILEKHLCSLEHELKAVNIRVATQQHLIEELQSK
NIELEEVCNACDVEKTLLLEKLHGMEELSTEYSILKKSFSNAIVEMEDLKEIVKELEASKN
SLKYDVSLHATEKDALALELETLGKRCADVLEEKSILETSFSNVNYELQELRVKYKDSEES
SRSYLADNTALLAEKHKLLSQLESTAVSLKFLEDKHADLRDNHGSLLSEKVLLCNQVNDY
EEMVSSLQDKIRHMDQMLEHEQQKCADASISTLILENSLVDARDKNLALFNECQKFIQAT
DSAEVLIAQLKEEARKEEEDKKALLNRNEKLRDGISEQIKVLNICKDLGPTDVVHDEIMLQ
TMSRETFNHVKHKEETEERNVFMDAELSVLGAILAQTVIGFRALHQQNCELVEEVESGAA
ELLFLQKENHKLIELNEQLEQRLQLGGNREEMLKIEILGLCKELSGLRQSYQTSQSEICNLT
KKYDSLLQEYKVLVEKYNALDDENAAVIAECIKLDLLSSFFHDLTVESASVLVSLDNDMA
MLSSVRHELDHEVTMLNRRAKILEMDFQHLKCTLENLLEALGSRLVLSEFDSNTSKIICQE
LTIECNSSMTQLMQKDDKLRKVDEKVQFLQERNQELCRVLRDLEVAVEDAEGVKVDLEK
KITTLTERGAVQDNETRLLREANNTLQVEVGIHEQKEESLMSTFETMRKEAEQHEREITLL
VCDTITRSVNTMVLEEQVLEMMMEREVLETRFFTEKDMLMKEISSRDAYVDDLQKRVAS
MRGENAGLMAELAAYLPLVASLSDQIRALEELEDGTLLLSELNKEGKLEFVQKDRHVPES
QDDSSGALKLRSLIARVEALHVVILDAKGRRDKEFTESVAQLEAANMEIQELKARKGSNA
KEECTEDDRQKYDADNSKGKHVQIMKDIELDQVSTCALYGTGATIYPLGGDANVELDDE
MLQLWETAERDCKNQTAKSSSSENDIQAVEEVKSEYPSFELARGRDPGIDRLEISAVSLEPQ
QLWSKNVLDKLASDAQRLSIVQASIEEIKQKMVGASKGKSTVSSEYSSIRAQLQEIDGSVL
EQIDFNCNVTKKAENYPAFEVSAELEGYSSRRKISEQVQKGSEKVAKLELELQKIQYVLLK
LEEEHEYKRVKAPEKRSRVLLRDYMTARKDKNDAGQKTKKKRIPFCGCVRIKSRTEP

>LOC_Os12g41200.1
MEMMSPTNPMRKYSWWWDSHISPKNSKWLQENLTDMDSKIKRMIKIIDEDADSFARRAE
MYYRRRPELMSLLEELYRAYRALAERHDHAAGELRSAQRKMAEAFPDEFQLDLDDDLPA
ETLSTETEADSRDMTPFFLSFINSGDSKKRAKDDQEHEKLQKEISSLSQENQELKKKISSVL
ENSDRAESEVASLKEALAQQEAEKEAAFSQCQQSSDRLQALKSEILQTQEEFKRLKEEMQ
NGLENLSTAEERCLLLERANQNLYSELDKLKNDSKERHGELNEKHVELEKLSISIQEEQLK
SMQAEMTRLSLEKQLAQAKEKLRILTLEKNGEASKFNDIEASKVRLQNDLDKIREENRKL
EEQNNSSISAIIRLQDEVISLKNAQRLLEEEVSRHVEEKKVLQYELSQLKDDKGDSERKHF
SIKEQIQVVNFNVESLQALAQEVRDGNVELKETIKHHEGVKALYVDNLMQLERTLERNA
HLERSLSAATTEVEELREKKVALEESCKHLNSKINGFQSERSMFIARIEGISHTMEKLSEKN
VFLENLLSENNTELEILRRKLNDSEESTHALLNQNSVLRSEKRTLVREVDSMNGALLNLEA
QFTELEGHHLDLQQEKNKASSEVIMLQEMLRLEREAHKELNYSGKTQFSAVQKQLSFLLE
EGRRRENQLQDEEHKIVEAQMEIFVLQKCLGDMAEANSDVSGQLQKQKELCEIQEEKLTF
LTENNQRLTEGIGSVMEELHLDDKYGSLDLMKLDVIVQLILHEIKCLLNTISDAQDVKQNQ
ILEKSLVVTLLEHFGREVADLRSERSVLRQEWQAKSEELLQLQNERHDLMKISCELRKEM
EARNRRVEEMKGEAKFLVRQLSELQESRQSLQAEVIKLIEENSSLSGKLYDSREKEKTAND
DFNTLLGEAISTDILGVVFKSLHDERTSQLQSLHEDFGSLHAAGNELYQEIKLMNKKLGDL
QLENNYLEKELSKTMSICDSSGSEIGAGRRRTMRRDTKLLKSGRKSQQESTVNIEQRKEID
HAGLEKSNELLREKLHKLQSEVQALRSKEQPVIDVKSCDAEITKLLTNMQMATANAALFK
EKVLELIASCESFEISEMVQKEVLKEEITRRNSYVNALKDKLNAVEIENSRLKVDLNGDFT
LLGALQTEVSALEKQTMSLAKDCLPSNKLRMEEFSVSPQLSKIAVKPIHGEPNATKMVKD
MELEKLHGTIKALQKVVTDTGVVLEQERLDFNANLLDARRQIDLLRLRDDMAAAVDDSD
AASDPAAAAYDRRLLKDIQLDLVQTTTPTNRSRAATATATAAASSQRHHRRRNGGSTEAPP

LGLWSVVRASRRRQQEEGGDGDDDDLRPPQSEASAERGRRSCSSEVSQLTVVKDLSVDK
QELLLPPRPPPPAMAEAPHREWKKKVIERLTADAQRLVDLQSIVGELRASAEAAPELDDVT
AQMVDAESAVAQLIDTNGKLLRKAEEFTSADAAGGAAGDDLRSRSQRKILERVRKMSEKI
ARLEQETQRFQHALLRHEEERATRRAAAAAATAAASSGKSSAAVQRRSSRVQLVEYLYGR
RRDSRRQRRGPSCCMRAKAIDD

>LOC_Os03g43684.1
MEETSPTNMRRKYSWWWNSHICPKNSKWLQENLTDMDSKIKMMIKIIEEDAESFAKRAE
MYYRRRPELMALLEELYRAYRALAERYDHAAGELRQAHRKIAEVFPEQVLVDLDDDLPA
ETASIETEMDNPDMAPYFLSFINASDSKKQAKDNQDNERLQKELESLSEENKDLKSRISSL
LEQTNKAELEVVCLKEALAQQNTEKEAIVLQCQQSTARLQNLKSEILHTQEKFNRLKEEM
QSGFQPFTTADERSVLLEKANQEMNLELNKLKHMLKQKHEELNEKQAELEKLNISTEEEH
LKCMQAEMAQLSLEKQLILAQDKMRLLALEKQIEVSKAKDTETEKVMLEKELEKIQKES
TSLNDQIHSSSSMIIRLQDEIITMKNAQRRLEEDVCRHVDEKKTLQNELCHLKEDRSDLDK
KHSSIKEQIQAVDLNVESLQALVQELKDGNVELKGIIRNHESTEVLHIENLRRLERMSEKN
SYLEKSLSAVTTELEVLREKKAELEESCKHLSSKISSHQSERAVLVAQIEAISQTMAELFEK
NVFLENSLSDANAELESLRGKLKELEESSEALYSQNSALQHEKSTLACQVDRISDTLQNLE
AHYAELEKRHSDLQEEKGSVLDEVIKLQEQIRFERKEHNDLEHSRKSQLDALHEKINVLS
QEGWNREEQLEEEEQNIVKAQTEIFIWKQCLEDIADANSDFLAQLKMKQEVCQVLEEKM
EYLSENNQKLTKCIGSVLKVLHLEEKYESLDQMKLDSIVHLILHEINCLLNTISDAQDVKQ
NELVEKSLVVTLLEHFGQEVADLRSERNTLKQEQQAKSEELLQLQREKQELVNITDEFWE
EVETRNRKVDELRAEAKFLVGQLSELQGSRRSLQSEIIKLIQENSMLSDELCDSREKERVFE
DDFSILISEVMSKDILSVVFRSLHEERTLQLVSLHSDFAQLQAAGSELYQDIKMMNMKLGD
LEKESNECNKELSRTISICNSTSTENAIGSGYPVGRDTDHLNSGRSQLEYHVNMETGEIEVD
MAGLEKSNEMLQEEVHKMQSEMEVLTSKENSAIDIKSCDEDIKRLLANMQMAIVNAALF
KEKVLELIITCESFEISSMVQKEVLKEEITRRNSYVDELKDKLNAVEIENRRLKVDLNGDFT
VLGSLQNEVSALEKQTLSLANDCLQSNKLRMEENALSTQVLKTNMRSSGDQNTVRTVKD
MELQKLHGTIKALQKVVTDTAVLLDQERLDFNANLQEARKQIEVLKLKEILDDDLIEMNY
EQMLKDIQLDLIQISSGNKTGSLGQANKTVAQANEKMLDSHGIVGASSSHVRNDLRPPQS
ESFERDNYKRPPSELMVVKELSIDKQELPRSITTEPHQEWKNKVIERLASDAQRLNALQSS
IQELKTNTEASEGLELESVRYQIREAEGFITQLIDSNGKLSKKAEEFTSEDGLDGDNIDLRS
RHQRKIMERARKMAEKIGRLEVEMQKVQEALLKYEEQTSTRTSKTMHRRSKVQLVDFLY
GRRRDSRKQQRCSPCGCMKANAIDD

>LOC_Os01g74510.1
MLQRAASNAYSWWWASHIRTKQSKWLDSHLQDMEHRVKCMLLLLGEEADSFSKRAEM
YYKRRPEVITQVEEVYRAYRGLADRYDIISGELHKANHTIATAFPDQVQYAMLEEEDDNIP
KAFTPVDPRKIHKSTVDGLMKKKKGGEQPAGSMNKNTTSAPIDKDNAREEISRLQKEILV
MQTEKEFIKSSYESGIAKYWDLEKQINDMQEQVCHFQDKFDESAVIEDDEARALMTATAL
KSCEDTIVKLQEQRKTSASQAMGESERVKVLREKLKAVMEGHGKSLPDSPDPCDKNVRK
NHGFEMEEVQHIKLGEFETQTVLEKIKEHFERDGSISVAEITEHIDELVNKVVDLELMVSSQ
SSQIDRLCRENSELESCLQSLEEENVSDPDKVNEKLKKLEEELVRVQALESCFHKDESTIRS
NFSEAISRLSGISEMLQSSEHGGVGGTLAVADGKEEEEDNDAGGIDDVAEPQVQTEAASD
DVDPAGKSTADVDPAGKSTATQEEAQAVDVGQEKAGGCSRERGSLVRLRHISSDDLGGCD

DEAPAAVDDPDGMRKQKKGQEGEGVEEEKKVILVAEYRALLEENKDAKRRLAEVEKTN
QECMHEIRSLRELLSSGSSEAGAAAAGGGGGGGDSSGGRRGHRRTPSYSLGHHRKQSLSS
ISRMIRMGSTIHEGDESEKVKAEELRLPAVATSSSPLENKLRKDIDTLLEENLEFWMKFSSS
LQRVQEFQRKHDELMQQLQPAATDGNSDTKQKQKQEQQLRALKTELQVWSEQNAMLR
GELQCRFAALCDVQEEITAALEQGGGGGEFTSYQAAKFQGEVLNMQQENNRVSDELQAG
QDHVKGLQAQIEKKLQHGGVTLPDADGPAAGAGAGATTPPPLPLTRVASKSKVPLQSFLFP
AKAKKPSLLARVTPVLQKQQPDLRFLAKLQPR

>LOC_Os01g07370.1
MKRMQRMPTRKSHSWWWDSHISPKNSKWLAENLEEMDKQVKDMLKLIEDEGDSFAKK
AEMYFERRPLLVTHVENFYRMYRALAERYDNVTGELRKNIPSSLQSQGSLSISESDSETQS
APPTPKPDSEETTPKQKRKPRAAGFDVFLGSGGSSDISKKGSDGSSSSSSESDSEVDELRED
NGDGSPFALNERIAELEDELQEAREKLEALEEKNTRCQCEKLEEKLKDSHSEISSLQKELE
GQLAHHDHEIEKCKKELEHVHEKYSHDKSTLETEIIKLQDIVKNFEGDLAKMSQEKLQLK
AQVKELEQASRSLDDSSAQIMKLQEIIKDLQRRLDNDSNEKKMLEERAIEFEQVRKELEGS
RTEVAELQATINNLKADLGRALEEKSQLESRINDLEHTIACNLEEFSQEKSSLGAEIQKLKE
ANASLEGKLTSTESQLQQLHAEKSEASISSEKQISDLNQAIADLETKLELLSSEKTTVDNKV
ASLLTDVTARDEKIREMDSHLHQLHLEHVKLIAEADAVTKAVSELRARVSELEEEVEEQKL
MVSDGAEGKREAIRQLCFSLEHYRHGYQQLRQLLQGHHKRPLVMAN

>LOC_Os01g07370.2
MKRMQRMPTRKSHSWWWDSHISPKNSKWLAENLEEMDKQVKDMLKLIEDEGDSFAKK
AEMYFERRPLLVTHVENFYRMYRALAERYDNVTGELRKNIPSSLQSQGSLSISESDSETQS
APPTPKPDSEETTPKQKRKPRAAGFDVFLGSGGSSDISKKGSDGSSSSSSESDSEVDELRED
NGDGSPFALNERIAELEDELQEAREKLEALEEKNTRCQCEKLEEKLKDSHSEISSLQKELE
GQLAHHDHEIEKCKKELEHVHEKYSHDKSTLETEIIKLQDIVKNFEGDLAKMSQEKLQLK
AQVKELEQASRSLDDSSAQIMKLQEIIKDLQRRLDNDSNEKKMLEERAIEFEQVRKELEGS
RTEVAELQATINNLKADLGRALEEKSQLESRINDLEHTIACNLEEFSQEKSSLGAEIQKLKE
ANASLEGKLTSTESQLQQLHAEKSEASISSEKQISDLNQAIADLETKLELLSSEKTTVDNKV
ASLLTDVTARDEKIREMDSHLHQLHLEHVKLIAEADAVTKAVSELRARVSELEEEVEEQKL
MVSDGAEGKREAIRQLCFSLEHYRHGYQQLRQLLQGHHKRPLVMAN

>LOC_Os01g61910.1
MLRRAASNAYSWWWASHIRTTQSKWLDNNVQEMEIRVKAMIKLIDIEADTFARKADLYF
KSRPDLINHVEETYRSYQALADRYDRVSGELHKSNHTIATAFPEQVQLSLQDDNGDGFPK
GITGININRGTSAAPKRTQTHKKISSKMSKDKAQEEIERLQKKILVLQTEKEFFKSSYESSLN
KYLSIERQAAEMQEEVWSLQETFSTSAVIEDNEARALMAAQALISCEDKLASLHCEQKRS
YEETTMEIQRVIDAKKKITIFKSECGYPDDQKDLPNHQDIEFSSIPSSIEDSDLIMKDCKLEL
QELSQKVKQKFESSSEASAVHLAGQVDEIVDKVISLEIAASSQNAQINRMKNEADELQKR
LDSLEDEKAALIEDSSKLSERLKQVEEVLQTIQRIGKSVHSENGNIHQQLTEVDDSLNDFV
KKLDAHSTDEIVNSSQDDSGIACKSKDEGLLDALDDSSKAHKEEPDETLGWQQLDLNGL
EDKDKILLKDYASILRNYKDTQKQLLEIEKRNREYHLEAMSEMNELKSSSATKDDEIRSLR
RMLSSLQTKLNNSLPQRFVESEESSEANASPSLENKNIAETEEYMKIREHEEPHDPSLEDKF
RAEISRILEENLDFWLRFSTSYHYMQKFQKSFDKAKAEMDKLTDAKAQEGSDAVPSCQSA

RKQESAVLEKKLRGLSTDLQVWLEKNVLLQGELESRFSLLCSIEEEISKITALGQTDEAHFT
PFQAAKFQGEVSSMKQENSKVTKELQTGMDHVRSLQVEVGRALLKLRENIELSIGRRNRT
QHSFRSLSMKAGVPLRTFLFGSKPKKASLFSCMGPVMPKPVADMRAGPFG

>LOC_Os05g39000.1
MLQRAASNAYSWWWASHIRTTQSKWLDTTLHEMEDRVKAMLNLIGADGDSFGKKAELY
FKSRPELINHVEEMFRSYQALADRYDRISSELHKANHTIATAFPDQIQFSMQDADGEGFQK
AISGIDLSNFKFPALEGLPMGSRGASRGTSPVPKRTQMHRRITSHMNKENAQEEIDKLQKQ
ILVLQTEKEFLKTSYDSALGRYLDIEKQVVELQDEVCSLQDAFSTGAAIEDNEARALMAA
RAIVSCEDTLVNLQDQQRRSSEEARTEFKRFIEAKKKLDTFKAECGQPHTQNDEPDNSDK
EYIHAMPSGDVDDSVQNEIRFDLQEVCQKVKELIELHPGVSVTDLADKVDRLVEKVIDLE
LATTSQNAQINRMKTEIDDLHKCLQALEEEKSALVADSSKLVDRLKQVEEVLQAVQHLGN
SIQNGTQNIHKEMNAACSELAEFVEKLHEPEPQNSGFMNSSQESSCQEEDSEVTSQYAKK
QTSDSIDGSKNEVEKQDKGSEGPLVQQHPDTNGSDGEDKILLEGYASVLQSYKGTEQKLS
EIEKTNQEYHSRSMSELKDLKSANAMKDEEIHSLRRMLSSLQRKMNAPAPENVDKSEETS
KISTTPVTEDKEIAEIEEYMKQCQVEEQLASSISEEKFRAEIDRVLENNLGFWLRFSTSYHQI
RNFQTSFDKLKTEMDKLIDAQAQCGADGVPISYQVAKLESAVLEKKFRDLNTDLQVWIEK
NVLLKGELENRFSSLCGIQEEISKIATLDKSDEVHFTPFQAAKLQGEVLNMKQENNKVAKE
LEAGLDHVRGLQVEVGRVLLKLRENLELSIARSNRAQQNFRNLSTKAGVPLRTFLFGTKP
KKPSLFSCMGPGVHKHHSGSRAGRR

>LOC_Os07g49480.1
MLQRAASNAYSWWWASHIRTTQSKWLDANLQEMETRVKIMLKLLGEEADTFGKRAEM
YYRRRPEVINHVEEVYRAYRALVERYDHLSKELHKANHTIATACPQHDVSLLQEQDDAEF
TPIRIQESTTTVQEVLNPKGHARPCAPHFTKQNAQQEIDTLQKAILVLQTEKEFVKSSYETG
ITKYREIEKQIADMQDQVCHIQNEFDAHASIEDDEARALMTITALRSCQGTVANLVKIFEEL
VRVAAGESEKVNYLRQKLYAMNYIIDPSKGEVGTTNIAVKNRVYPNTQEILELQPIYEKIEK
FFELNSESVVQEMAERVDELVDKVMNLELKFPKQSAQIKQLKEDNDSLKDRLDDLQDEIA
LRDDPSDLSEQLKLAEDELNRVKALERSVIEEEVLVSTVFSEVVSCITNISKAFGSIDPEDM
TSLSAAVENDGEITSDDISTSLPEEEFRGTEEATTDDNLGRDRCRKEDASGVEGHDSLDGT
DGIDDCKNGNEENFQSENRLIQEDLMDKRSIQASNNIDRIVIPGKENGFNNACEGKIDCSPS
GNTKKYRDIGNDVIDNSVQGESLKGEHPPTVISQTHLPHSECLDTLTNKSDSDEKGSSVVV
TVNSFGGSKRIQGLRIGGDENSMSGNSLIQEELRDDKSLKTPGYVNLVGSTNQHSLSDGST
TEEISLPKVSNSCFSDADMRLELCHTEEAISVEEWPKQDGQLIAPETMKSLNGGSKVDSSE
KGGRTSLEHMNSIQDLKTSELVDAHSSRVYQQVPKVTTKSNNIASCIPHGELEMRSSDGRE
HTRQASTLSKPGSMSLRVNSSLVAERDAPSWQEFLLDGIEGREALLLDDYTLILRNYKETK
RRLAELEKKNEQHPKETKTVIRELRNANSMKYVEIQSLRDLLDPSEDISSTHSKMGFNRSN
HPLDTEISVLEGIDVRHTGVRKNTSPFEVKFRSEIDALVEENLQFLVRFSMACHRMQDFDS
KYQELQKGMGDFEVKKTGEPDAAAESDPAEKKLRELRTELDVWFEQNALLDQDLQLKT
MSLCRLQEEIAEALRASAETDGGRFTPYEAAKFQGEVLNMQQSSGKIERELQSALKRMRE
LEGKVNDGLQKLRESFDLSCRRSSLVEEESSSTSYHSQFKHFPTRTRVPLRNFLFGTKPKK
KSIFACINPTLQKQFSDL
